# Proteomic differences between focal and diffuse traumatic brain injury in human brain tissue

**DOI:** 10.1038/s41598-018-25060-0

**Published:** 2018-05-01

**Authors:** Sami Abu Hamdeh, Ganna Shevchenko, Jia Mi, Sravani Musunuri, Jonas Bergquist, Niklas Marklund

**Affiliations:** 1 Department of Neuroscience, Neurosurgery, Uppsala University, Uppsala, Sweden; 2 Analytical Chemistry, Department of Chemistry-BMC, Uppsala University, Uppsala, Sweden; 3 Medicine and Pharmacy Research Center, Binzhou Medical University, Yantai, China

## Abstract

The early molecular response to severe traumatic brain injury (TBI) was evaluated using biopsies of structurally normal-appearing cortex, obtained at location for intracranial pressure (ICP) monitoring, from 16 severe TBI patients. Mass spectrometry (MS; label free and stable isotope dimethyl labeling) quantitation proteomics showed a strikingly different molecular pattern in TBI in comparison to cortical biopsies from 11 idiopathic normal pressure hydrocephalus patients. Diffuse TBI showed increased expression of peptides related to neurodegeneration (Tau and Fascin, *p* < 0.05), reduced expression related to antioxidant defense (Glutathione S-transferase Mu 3, Peroxiredoxin-6, Thioredoxin-dependent peroxide reductase; *p* < 0.05) and increased expression of potential biomarkers (e.g. Neurogranin, Fatty acid-binding protein, heart *p* < 0.05) compared to focal TBI. Proteomics of human brain biopsies displayed considerable molecular heterogeneity among the different TBI subtypes with consequences for the pathophysiology and development of targeted treatments for TBI.

## Introduction

Traumatic brain injury is a global health problem and a leading cause of death and severe disability^1,2^. Importantly, TBI is not one single event but a disease process beginning at time of impact, and then exacerbated by a complicated series of secondary insults that may continue many years after the initial injury^2^. Although the initiating molecular events are incompletely known, the initial impact offsets a cascade of biological pathways at the cellular level which include influx of calcium ions, mitochondrial damage and increase in free radical production leading to major disturbances in energy metabolism and extensive damage to the cytoskeleton^3,4^.

TBI may be “the most complex disorder in the most complex organ in the body”^5^, since it has a marked heterogeneity displayed as different types of brain injuries, unified by brain damage initiated by an external force^1^. TBI is commonly categorized into either focal injury that includes subdural, epidural and intracranial hemorrhages, or diffuse injury with widespread damage to axons. Diffuse axonal injury (DAI) is associated with angular or rotational acceleration-deceleration forces to the head resulting in shear, tensile, and compressive strains of brain tissue^6,7^. Shearing of axons rapidly leads to disruption of axonal transport and axonal swelling, which may lead to secondary disconnection^8^. Axonal injury, found to progress years post-injury, may increase the risk of neurodegeneration including Alzheimer’s disease-like pathology, such as extracellular insoluble aggregates of beta-amyloid (Aβ) peptides, and accumulations of hyperphosphorylated tau (HPtau) into neurofibrillary tangles (NFT)^9,10^.

Despite an extensive number of clinical and experimental publications evaluating pharmacological modulation of the secondary injury cascades, there are currently no drug therapies with proven clinical efficacy for TBI^11,12^. One major reason for the failure of these clinical studies is the inclusion of heterogeneous TBI subtypes^13^, which constitute a major challenge in the search for novel pharmacological treatments. The currently used TBI classifications remain inadequate in appreciating the diversity of TBI. Therefore, further understanding of the different biological pathways associated with human TBI *in vivo* is warranted.

We hypothesized that there may be fundamental molecular differences between focal TBI and DAI in humans. In this report, mass-spectrometry (MS)-based proteomics were used on fresh brain biopsies of structurally normal-appearing frontal cortex, obtained in conjunction with the insertion of an intracranial pressure monitor acutely in 16 severe TBI patients. Eleven cortical biopsies from patients with idiopathic normal pressure hydrocephalus (iNPH) were also similarly obtained from the frontal region and served as controls. The aim of the study was to investigate potential differences in protein expression in focal and diffuse injury in the acute phase after TBI. Our findings show that DAI initiates unique biological pathways in comparison to focal TBI, with regulatory differences in proteins involved in energy metabolism, cytoskeletal functions, and mechanisms of oxidative stress as well as differences in the regulation of proteins suggested to have important roles in the development of neurodegenerative diseases.

## Results

### Patient characteristics

**Figure 1:**
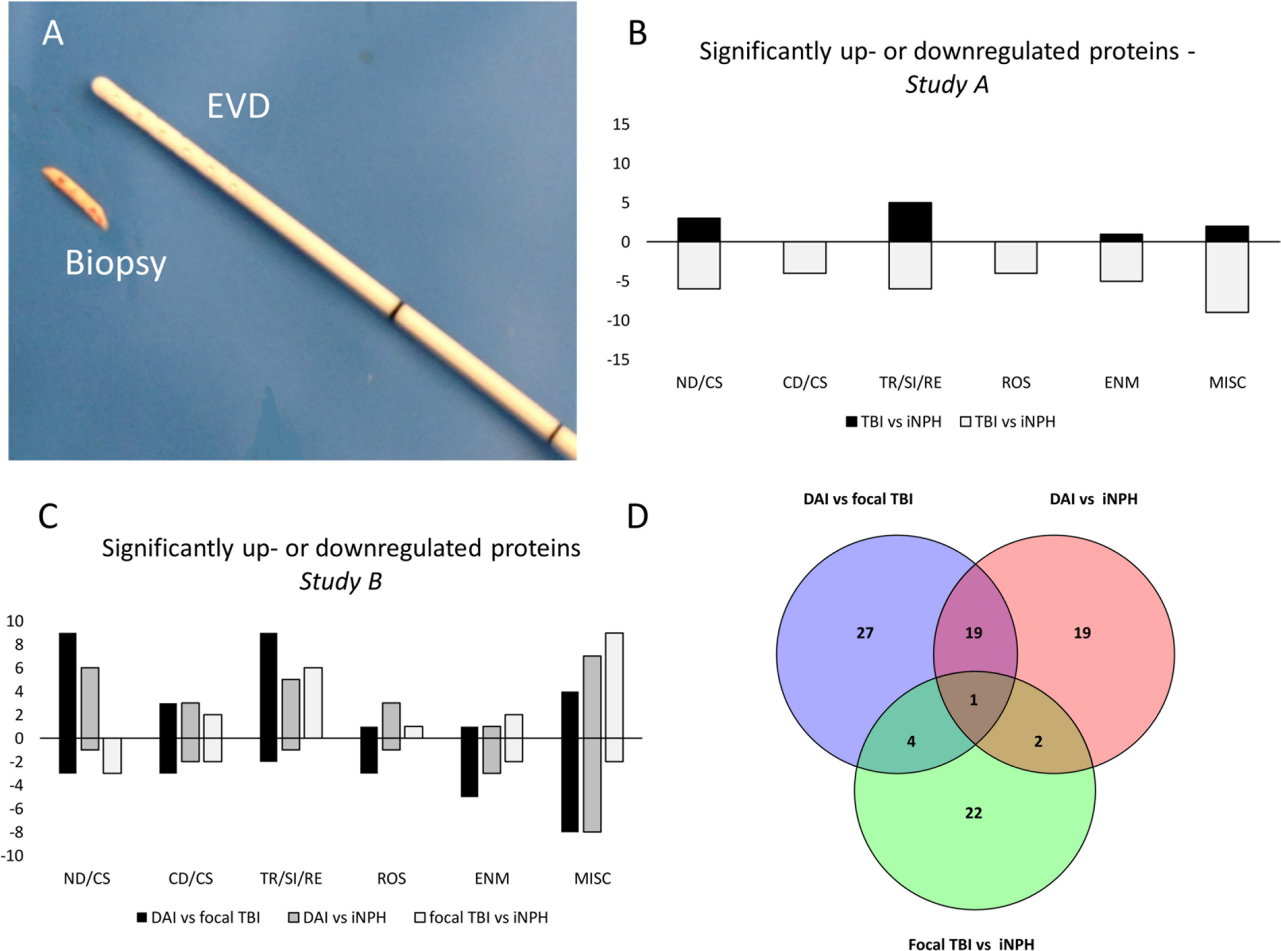
(**A**) Tissue biopsy, obtained with a biopsy needle with 14 gauge, 2.11 mm outer diameter and an 8 mm side cutting window (Elekta Instrument AB, Innsbruck, Austria), to be compared with external ventricular drain (outer diameter 2.5 mm) placed into the same cortical area at site of biopsy. (**B**) Diagram demonstrating differentially expressed proteins in patients with severe traumatic brain injury (TBI) when compared to patients with idiopathic normal pressure hydrocephalus (iNPH) (*Study A*). Positive values represent upregulated proteins. Negative values represent downregulated proteins. For a detailed list of differentially expressed proteins see Table 3. (**C**) Diagram demonstrating number of differentially expressed proteins among groups in different cellular mechanisms in *Study B*. Positive values represent upregulated proteins. Negative values represent downregulated proteins. For a detailed list of differentially expressed proteins see Tables 4–6. (**D**) Venn diagram demonstrating overlap in differentially expressed proteins in the analysis of diffuse axonal injury (DAI) vs focal TBI, DAI vs iNPH and focal TBI vs iNPH. For a detailed list of differentially expressed proteins with overlap see Suppl. Table 2. EVD = external ventricular drainage, TBI = traumatic brain injury, iNPH = idiopathic normal pressure hydrocephalus, DAI = diffuse axonal injury, ND/CS = neurodegeneration/cytoskeletal, CD/CS = cell death/cell survival, TR/SI/RE = transport/signaling/repair, ROS = reactive oxygen species, ENM = energy metabolism, MISC = miscellaneous.

Sixteen severe TBI patients, defined as post resuscitation Glasgow Coma Scale (GCS) score ≤ 8, were conveniently recruited. Detailed demographic and clinical characteristics are shown in Tables 1 and 2. The mean age of TBI patients (12 males, 4 females) was 43.7 ± 20.7 years and the mean age of iNPH patients (7 males, 4 females) was 73.7 ± 5.2 years (*p* < 0.0001). The tissue samples, obtained in conjunction with an intracranial pressure (ICP) monitor insertion (Fig. 1A), were included in two separate proteomics analyses. In the first analysis (*Study A*), the objective was to analyse whether molecular differences existed in structurally normal-appearing cortex between TBI and iNPH and therefore six TBI patient with combined diffuse and focal TBI were compared to six iNPH patients for proteome differences. In the second analysis (*Study B*), differences between TBI subtypes were evaluated and thus, five patients with DAI, five patients with focal TBI and five iNPH patients were compared for proteome differences (Tables 1 and 2 and Suppl. Figure 1).

**Table 1:** Patient characteristics in *STUDY A and B*.

Patient#	Age	Gender	Cause of injury	Other injuries	CT Marshall	Main TBI lesion	Biopsy time post-injury (h)	Location of biopsy	mGCS at admission	mGCS at discharge
TBI (*STUDY A*)										
#1	44	M	MVA	Thi	DI I	DAI (**-**MRI)	4	RF	3	6
#2	20	M	MVA	None	DI II	DAI III	7	RF	5	6
#3	62	M	Fall	None	NEM	aSDH, Ccx	8	RF (CL)	5	6
#4	73	M	MVA	Ffx	NEM	Ccx	48	RF (IL)	4	6
#5	17	F	BcA	Ffx	DI II	DAI (-MRI)	14	RF	5	6
#6	38	M	MVA	Ffx	DI II	DAI II	3	RF	4	6
DIFFUSE TBI (*STUDY B*)										
#1	21	M	MVA	Thi; Sfx	DI II	DAI II	11	RF	5	6
#2	18	M	MVA	Thi	DI III	DAI III	8	RF	4	5
#3	23	M	MVA	Ffx	DI II	DAI III	4	RF	4	6
#4	39	M	MVA	Thi	DI III	DAI III	10	RF	3	1
#5	40	M	MVA	Ffx; Sfx	DI III	DAI I	16	RF	4	6
FOCAL TBI (*STUDY B*)										
#1	56	F	MVA	None	NEML	aSDH	12	LF (CL)	3	5
#2	44	F	N/A	None	NEML	Ccx	59	RF (CL)	5	6
#3	59	M	Fall	Ffx	NEML	Ccx	10	RF (CL)	5	6
#4	62	M	Fall	None	NEML	Ccx	175	RF (IL)	5	5
#5	83	F	N/A	None	NEML	aSDH	32	LF (IL)	5	5

M = male; F = female; mGCS; motor component of the Glasgow Coma Scale; MVA = motor-vehicle accident; BcA = bicycle accident; N/A = not established; Thi = thoracic injury; Ffx = facial fracture; Sfx = spinal fracture; DI = diffuse injury; NEML = non-evacuated mass lesion; DAI = diffuse axonal injury; aSDH = acute subdural hematoma; Ccx = Cortical contusion; - MRI = MRI not available; TBI = traumatic brain injury; RF = right frontal; LF = left frontal; CL = contralateral to focal lesion; IL = ipsilateral to focal lesion.

**Table 2:** Patient characteristics in the evaluated cohorts of idiopathic normal pressure hydrocephalus (iNPH) patients.

Patient #	Age (years)	Gender	MMSE	Location of biopsy	CSF Aβ1–42 (ng/L)	CSF tau (ng/L)	CSF Hptau (ng/L)	Neuropathology
iNPH (*STUDY A*)								
#1	78	M	16	RF	759	223	24	Gliosis, Hptau
#2	80	F	14	RF	416	236	33	Aβ aggregates
#3	64	F	30	RF	887	370	48	Gliosis
#4	75	F	29	RF	N/A	N/A	N/A	Gliosis
#5	76	M	24	RF	541	115	19	Gliosis
#6	77	M	21	RF	448	206	36	Aβ aggregates
iNPH (*STUDY B)*								
#1	67	M	25	RF	673	197	29	Gliosis
#2	80	F	22	LF	756	112	19	Gliosis
#3	72	M	27	RF	639	204	28	No pathology
#4	71	M	25	RF	365	130	18	Hptau
#5	71	M	17	RF	498	99	24	No pathology

Reference interval: CSF-Aβ1-42 > 550 ng/L, CSF-Tau < 400 ng/L, CSF-HPtau-tau < 80 ng/L, M = male; F = female; RF = right frontal; LF = left frontal; N/A = not established; MMSE = preoperative mini-mental state examination; CSF = cerebrospinal fluid; Aβ = Amyloid-beta aggregates; Hptau = hyperphosphorylated tau.

In *Study A*, patients with TBI (mean 42.3 ± 22.3 years) were younger than patients with iNPH (mean 75 ± 5.7 years, *p* = 0.006). In *Study B* patients with DAI (mean age 28.2 ± 10.5 years) were younger than patients with focal TBI (mean age 60.8 ± 14.2 years, *p* = 0.003) and patients with iNPH (mean age 72.2 ± 4.8 years, *p* < 0.0001). No significant age difference was found between patients with focal TBI and patients with iNPH. The biopsy was obtained 26.3 ± 42.8 hours post-injury, which did not differ significantly between *Study A* and *B* or between DAI and focal TBI patients in *Study B*.

One TBI patient had a 1.1 ml hematoma on post-operative CT scan at the biopsy/ICP monitoring site of no clinical significance. In iNPH patients, no adverse events were observed after the collection of brain biopsies.

Label free (LF) and a stable isotope dimethyl labelling (DML) shotgun based bottom-up approaches were used for the detection and quantification of the proteins in both *Study A* and *Study B*. These two methods are complementary to each other and have different inherent biases. The LTQ-Orbitrap mass spectrometer was used only in *Study B*, while nano-LC-MS/MS experiments were performed using 7 T hybrid LTQ-FT mass spectrometer in both *Study A* and *B*. The experimental analyses were also performed at two different time points why we decided to present the data from the two studies separately.

### Proteome differences in a cohort of TBI and iNPH patients (Study A)

LC-MS/MS analysis using LF and DML proteomics approaches identified a total number of 692 unique proteins. Among these, 316 proteins found in at least 5 out of 6 patient pairs were subjected to statistical analysis. Of these, 45 proteins were found to be significantly increased (n = 11) or decreased (n = 34) in TBI in comparison to iNPH (Table 3). Of the significantly up- or downregulated proteins, 20% (n = 9, increased n = 3, decreased n = 6) are involved in neurodegeneration or cytoskeletal function, 9% (n = 4, increased n = 0, decreased n = 4) in cell death or survival functions, 24% (n = 11, increased n = 5, decreased n = 6) in cell signaling, transport or repair pathways, 9% (n = 4, increased n = 0, decreased n = 4) in oxidation/reduction pathways and 11% (n = 6, increased n = 1, decreased n = 5) in energy metabolism. The remaining 24% (n = 11, increased n = 2, decreased n = 9) are involved in a variety of cell functions including neurite outgrowth, protein metabolism and regulation of nucleic acid (Fig. 1B). The analysis identified 2 immunoglobulins, both downregulated by TBI in comparison to iNPH (Table 3

**Table 3:** Mass spectrometry analysis of brain tissue biopsies from structurally normal-appearing frontal cortex- *STUDY A*.

Biological process	Gene name	Protein name	Number of platforms	Ratio	↑↓	IP	P-value	Reference*
**Neurodegeneration/Cytoskeleton**	CADM2	Cell adhesion molecule 2	2	0.526193	↑	2	0.0103	(*1*)
	TBB2A	Tubulin beta-2A chain	2	0.331083	↑	7	0.0238	(*2, 3*)
	TBB5	Tubulin beta chain	2	0.164451	↑	2	0.0198	(*3, 4*)
	SPTA2	Spectrin alpha chain, non-erythrocytic 1	2	−0.07552	↓	38	0.0268	(*5–7*)
	SPTB2	Spectrin beta chain, non-erythrocytic 1	2	−0.107	↓	25	0.0091	(*8–10*)
	GFAP	Glial fibrillary acidic protein	2	−1.02862	↓	19	0.0269	(*11*)
	MARCKS	Myristoylated alanine-rich C-kinase substrate	2	−0,23777	↓	2	0,0414	(*12, 13*)
	VIME	Vimentin	2	−0,77168	↓	2	0,0080	(*14, 15*)
	EPB41L3	Band 4.1-like protein 3	2	−0.31153	↓	4	0.0047	(*16*)
**Cell death/survival**	UBE2N	Ubiquitin-conjugating enzyme E2 N	2	−0.10842	↓	2	0.0342	(*17, 18*)
	YWHAE	14-3-3 protein epsilon	2	−0.26886	↓	5	0.0132	(*19–21*)
	UBA1	Ubiquitin-like modifier-activating enzyme 1	2	−0,43635	↓	2	0,0006	(*22*)
	CRYAB	Alpha-crystallin B chain	2	−1,28166	↓	3	0,0371	(*23*)
**ROS/RNS**	MDHC	Malate dehydrogenas, cytoplasmic	2	−0.12386	↓	7	0.0442	(*24*)
	PRDX6	Peroxiredoxin-6	2	−0.55867	↓	3	0.0357	(*25, 26*)
	PARK7	Protein DJ-1	2	−0,24841	↓	4	0,010	(*27–29*)
	PRDX2	Peroxiredoxin-2	2	−0,36942	↓	5	0,0351	(*30, 31*)
**Transport/Signaling/Repair**	RAB3A	Ras-related protein Rab-3A	2	0,82717	↑	4	0,0402	(*32*)
	SYPH	Synaptophysin	2	1,502137	↑	2	0.0448	(*33, 34*)
	SYN1	Synapsin-1	2	0.938617	↑	11	0.0384	(*35, 36*)
	SCRN1	Secernin-1	2	−0.1778	↓	2	0.0271	(*37–39*)
	REEP5	Receptor expression-enhancing protein 5	2	0.603423	↑	2	0.009	(*40*)
	4F2	4F2 cell-surface antigen heavy chain	2	0.427954	↑	2	0.0311	(*41*)
	CNTN1	Contactin-1	2	−0.15205	↓	4	0.0361	(*42, 43*)
	PEBP1	Phosphatidylethanolamine-binding protein 1	2	−0,25765	↓	6	0,0352	(*44*)
	TRFE	Serotransferrin	2	−0,72002	↓	4	0,0293	(*45, 46*)
	HBA	Hemoglobin subunit alpha	2	−0,84398	↓	6	0,0500	(*47*)
	CAH2	Carbonic anhydrase 2	2	−0,85028	↓	3	0,0288	(*48*)
**Energy**	PKM	Pyruvate kinase	2	−0.21203	↓		0.0078	(*49, 50*)
	ATP5J	ATP synthase-coupling factor 6	2	0.471757	↑	2	0.037	(*51*)
	ALDOC	Fructose-bisphosphate aldolase C	2	−0.24447	↓	9	0.0219	(*52, 53*)
	NSE	Neuron specific enolase	2	−0,09553	↓	9	0.0343	(*54*)
	LDHB	L-lactate dehydrogenase B-chain	2	−0.21642	↓	7	0.0114	(*55*)
	LDHA	L-lactate dehydrogenas A chain	2	−0.17399	↓	6	0.0272	(*56*)
**Miscellaneous**								
**Neuronal outgrowth**	NRCAM	Neuronal cell adhesion molecule	2	0.389567	↑	2	0.0462	(*57*)
	IGSF8	Immunoglobulin superfamily member 8	2	−0,31939	↓	3	0,0309	(*58, 59*)
	LSAMP	Limbic system-associated membrane protein	2	−0,55395	↓	2	0,0229	(*60*)
**Regulation of nucleic acid metabolism**	HPRT	Hypoxanthine-guanine phosphoribosyltransferase	2	−0.16494	↓	2	0.0138	(*61*)
**Immune response**	IGHG1	Ig gamma-1 chain C region	2	−0.65507	↓	3	0.0203	(*62, 63*)
	IGKC	Ig kappa chain C region	2	−1,1249	↓	2	0,0182	(*64*)
**Protein metabolism**	HS90A	Heat shock protein HSP 90-alpha	2	−0,20697	↓	13	0,0121	(*65*)
	ALDH6A1	Methylmalonate-semialdehyde dehydrogenase	2	−0.34609	↓	2	0.0103	*(66)*
	VPP1	V-type proton ATPase 116 kDa subunit a	2	0.591705	↑	4	0.0147	(*67, 68*)
	DDAH1	N(G)-dimethylarginine dimethylaminohydrolase	2	−0.15192	↓	2	0.0302	(*69, 70*)
**Lysosomal and peroxisomal degradation**	PSAP	Prosaposin	2	−0.69792	↓	3	0.0050	(*71–73*)

Significantly up- or downregulated proteins in a cohort of severe traumatic brain injury (TBI) patients in comparison to patients with idiopathic normal pressure hydrocephalus (iNPH).

IP = Number of identified peptides, ↑↓ = Increased or decreased, * = Reference provided in supplementary material.

).

### Proteome differences between diffuse axonal injury (DAI) and focal TBI patients (Study B)

LTQ-FT MS and LTQ-Orbitrap MS analysis using LF and DML proteomics identified a total of 1844 unique proteins. Of these, 51 proteins were significantly increased (n = 27) or decreased (n = 24) in DAI. A list of significantly increased or decreased proteins in DAI when compared to focal TBI is provided in Table 4. Of the significantly up- or downregulated proteins 24% (n = 12, increased n = 9, decreased n = 3) are involved in neurodegeneration or cytoskeletal function, 12% (n = 6, increased n = 3, decreased n = 3) in cell death or survival functions, 22% (n = 11, increased n = 9, decreased n = 2) cell signaling, transport or repair pathways, 8% (n = 4, increased n = 1, decreased n = 3) in oxidation/reduction pathways and 12% (n = 6, increased n = 1, decreased n = 5) in energy metabolism. Of the remaining 24% (n = 12, increased n = 4, decreased n = 8), identified proteins were involved in neurite outgrowth, protein metabolism, regulation of nucleic acid and lysosomal function (Fig. 1C). One protein, microtubule-associated protein tau (MAPT) was identified in all four MS platforms as significantly increased in DAI. Pyruvate kinase (PKM), Cathepsin D (CTSD) and Prosaposin (PSAP) were identified in 3/4 MS platforms as significantly decreased in DAI when compared to focal TBI (Table 4

**Table 4:** Mass spectrometry analysis of brain tissue biopsies from structurally normal-appearing frontal cortex- *STUDY B*.

Biological process	Gene name	Protein name	Number of platforms	Ratio	↑↓	IP	P-value	Reference*
**Neurodegeneration/Cytoskeleton**	MAPT	Microtubule- associated protein tau	4	0.28–0.55	↑	25	0.02–0.0005	(*74–81*)
	MAP6	Microtubule associated protein 6	2	0.30–0.48	↑	37	0.0056–0.0046	(*82, 83*)
	FSCN1	Fascin	1	0.98	↑	31	0.047	(*84*)
	DMTN	Dematin	1	0.35	↑	13	0.024	(*85, 86*)
	TPM1	Tropomyosin alpha-1 chain	1	0.47	↑	14	0.021	(*87*)
	ADD3	Gamma-adducin	1	−0.71	↓	16	0.038	(*88*)
	GFAP	Glial fibrillary acidic protein	1	−0.88	↓	38	0.047	(*11*)
	CRMP1	Dihydropyrimidinase-related protein 1	1	0.39	↑	27	0.0004	(*89*)
	MAP1A	Microtubule-associated protein 1A	1	0.19	↑	99	0.0054	(*90*)
	EPB41L3	Band 4.1-like protein 3	1	−0.14	↓	51	0.011	(*16*)
	MAP1B	Microtubule-associated protein 1B	1	0.14	↑	98	0.048	(*90*)
	CAPZB	F-actin-capping protein subunit beta	1	0.25	↑	14	0.019	(*91, 92*)
**Cell death/survival**	YWHAB	14-3-3 protein beta/alpha	2	0.30–0.68	↑	17	0.049–0.0044	(*19–21*)
	PPP3R1	Calcineurin subunit B type 1,	1	0.84	↑	11	0.026	(*93, 94*)
	YWHAQ	14-3-3 protein theta	1	−0.14	↓	19	0.0499	(*19–21*)
	YWHAE	14-3-3 protein epsilon	1	−0.095	↓	26	0.017	(*19–21*)
	CD47	Leukocyte surface antigen CD47	1	0.18	↑	4	0.042	(*95–97*)
	CDC42	Cell division control protein 42 homolog	1	−0.25	↓	11	0.044	(*98*)
**ROS/RNS**	CRYM	Ketamine reductase	1	0.33–0.86	↑	16	0.030–0.0056	(*99–101*)
	PRDX6	Peroxiredoxin-6	2	−0.49	↓	22	0.022	(*25, 26*)
	GSTM3	Glutathione S-transferase Mu 3	1	−1.51	↓	14	0.0016	(*102, 103*)
	PRDX3	Thioredoxin-dependent peroxide reductase	1	−0.33	↓	10	0.044	(*104*)
**Transport/Signaling/Repair**	FABP3	Fatty acid-binding protein, heart	2	0.29–0.53	↑	11	0.022–<0.0001	(*105, 106*)
	AP2A1	AP-2 complex subunit alpha-1	1	0.54	↑	36	0.018	(*37*)
	DBNL	Drebin-like protein	1	0.58	↑	9	0.048	(*107, 108*)
	NRGN	Neurogranin	1	0.42	↑	3	0.0018	(*109*)
	SCRN1	Secernin-1	1	0.14	↑	19	0.015	(*37–39*)
	VSNL1	Visinin-like protein 1	1	0.11	↑	17	0.021	(*110, 111*)
	GNB1	Guanine nucleotide-binding protein beta-1	1	−0.11	↓	17	0.024	(*112*)
	NCALD	Neurocalcin-delta	1	0.32	↑	12	0.01	(*113, 114*)
	SEP_11	Septin-11	1	0.36	↑	19	0.03	(*115*)
	CLTA	Clathrin light chain A	1	0.28	↑	8	0.021	(*116, 117*)
	CNTNAP1	Contactin-associated protein 1	1	−0.59	↓	35	0.041	(*118, 119*)
**Energy**	PKM	Pyruvate kinase	3	−0.11–(−0.28)	↓	41	0.018–0.033	(*49, 50*)
	PYGB	Glycogen phosphorylase, brain form	2	−0.31–(−0.49)	↓	40	0.0028–0.0064	(*120, 121*)
	ALDOC	Fructose-bisphosphate aldolase C	2	−0.13–(−0.21)	↓	29	0.034–0.0028	(*52, 53*)
	NSE	Neuron specific enolase	2	−0.18–(−0.27)	↓	25	0.044–0.021	(*54*)
	LDHB	L-lactate dehydrogenase B-chain	1	0.61	↑	24	0.044	(*55*)
	TKT	Transketolase	1	−0.27	↓	37	0.0499	(*122*)
**Miscellaneous**								
**Neuronal outgrowth**	NCAM1	Neural cell adhesion molecule-1	2	−0.18–(−0.15)	↓	27	0.034–0.0028	(*123–125*)
	NFASC	Neurofascin	1	−0.24	↓	36	0.042	(*126*)
	TNR	Tenascin-R	1	0.14	↑	38	0.03	(*127*)
**Regulation of nucleic acid metabolism**	HNRNPA2B1	Heterogeneous nuclear ribonucleoprotein A2/B1	2	−0.15–(−0.20)	↓	16	0.018–0.002	(*128*)
	CMPK1	UMP-CMP kinase	1	0.53	↑	10	0.048	(*129*)
	GDA	Guanine deaminase	1	0.36	↑	22	0.031	(*130*)
	HIST1H2BN	Histone H2B type 1	1	−1.89	↓	7	0.026	(*131*)
**Protein metabolism**	CTSD	Cathepsin D	3	−0.49–(−0.94)	↓	14	0.039–0.00004	(*132, 133*)
	ALDH6A1	Methylmalonate-semialdehyde dehydrogenase	1	−0.37	↓	25	0.0086	(*66*)
	CALR	Calreticulin	1	−0.84	↓	15	0.0016	(*134–136*)
**Lysosomal and peroxisomal degradation**	PSAP	Prosaposin	3	−0.10–(−0.96)	↓	13	0.003–0.0006	(*71–73*)
	PHYHIP	Phytanoyl-CoA hydroxylase-interacting protein	1	0.31	↑	13	0.026	(*137*)

Significantly up- or downregulated proteins in biopsies from patients with diffuse axonal injury (DAI) in comparison to focal brain injury.

IP = Number of identified peptides, ↑↓ = Increased or decreased, * = Reference provided in supplementary material.

).

### Proteome differences between diffuse axonal injury and iNPH patients (study B)

LTQ-FT MS and LTQ-Orbitrap MS analysis using LF and DML proteomics identified a total of 1844 unique proteins. Of these, 41 proteins were significantly increased (n = 25) or decreased (n = 16) in DAI when compared to iNPH (Table 5). Of the significantly up- or downregulated proteins 17% (n = 7, increased n = 6, decreased n = 1) are involved in neurodegeneration or cytoskeletal function, 12% (n = 5, increased n = 3, decreased n = 2) in cell death or survival functions, 14% (n = 6, increased n = 5, decreased n = 1) cell signaling, transport or repair pathways, 10% (n = 4, increased n = 3, decreased n = 1) in oxidation/reduction pathways and 10% (n = 4, increased n = 1, decreased n = 3) in energy metabolism. Of the remaining 36% (n = 15, increased n = 7, decreased n = 8), identified proteins are involved in neurite outgrowth, protein metabolism, regulation of nucleic acid, immune response and lysosome function (Fig. 1C). MAPT and Triosephosphate isomerase (TPI1) were identified in 3/4 MS platforms as significantly increased in DAI. PSAP was identified in all four MS platforms while PKM and CTSD were identified in 3/4 MS platforms as significantly decreased in DAI when compared to iNPH (Table 5

**Table 5:** Mass spectrometry analysis of brain tissue biopsies from structurally normal-appearing frontal cortex-*STUDY B*.

Biological process	Gene name	Protein name	Number of platforms	Ratio	↑↓	IP	P-value	Reference*
**Neurodegeneration/Cytoskeleton**	MAPT	Microtubule- associated protein tau	3	0.23–0.33	↑	25	0.045–0.0021	(*74–81*)
	MAP6	Microtubule associated protein 6	1	0.51	↑	37	0.016	(*82, 83*)
	MAPT1	Microtubule- associated protein tau	1	0.70	↑	24	0.019	(*74–81*)
	DMTN	Dematin	1	0.36	↑	13	0.028	(*85, 86*)
	CRMP1	Dihydropyrimidinase-related protein 1	1	0.33	↑	27	0.0011	(*89*)
	MAP1A	Microtubule-associated protein 1A	1	0.12	↑	99	0.038	(*90*)
	NEFH	Neurofilament heavy polypeptide	1	−1.01	↓	31	0.018	(*138, 139*)
**Cell death/survival**	YWHAB	14-3-3 protein beta/alpha	1	0.23	↑	17	0.023	(*19–21*)
	YWHAZ	14-3-3 protein zeta/delta	1	0.15	↑	23	0.047	(*19–21*)
	NPEPPS	Puromycin-sensitive aminopeptidase	1	−0.63	↓	40	0.034	(*140*)
	BCAN	Brevican core protein	1	0.43	↑	28	0.042	(*141*)
	UBB	Ubiquitin B	1	−0.31	↓	5	0.0053	(*142, 143*)
**ROS/RNS**	CRYM	Ketamine reductase	2	0.34–0.42	↑	16	0.025–0.0017	(*99–101*)
	PRDX6	Peroxiredoxin-6	1	−0.48	↓	22	0.043	(*25, 26*)
	GSTO1	Glutathione S-transferase omega-1	1	0.71	↑	19	0.011	(*144, 145*)
	GGCT	Gamma-glutamylcyclotransferase	1	1.04	↑	5	0.020	(*146*)
**Transport/Signaling/Repair**	FABP3	Fatty acid-binding protein, heart	1	0.26	↑	11	0.015	(*105, 106*)
	AP2A1	AP-2 complex subunit alpha-1	1	−0.81	↓	36	0.048	(*37*)
	NRGN	Neurogranin	1	0.54	↑	3	0.00037	(*109*)
	SCRN1	Secernin-1	1	0.16	↑	19	0.022	(*37–39*)
	NCALD	Neurocalcin-delta	1	0.23	↑	12	0.018	(*113, 114*)
	ENSA	Alpha-endosulfine	1	0.71	↑	7	0.021	(*147*)
**Energy**	PKM	Pyruvate kinase	3	−0.26–(−0.23)	↓	41	0.040–0.00015	(*49, 50*)
	ALDH2	Aldehyde dehydrogenase	1	−0.20	↓	26	0.041	(*148, 149*)
	TPI1	Triosephosphate isomerase	3	0.14–0.37	↑	17	0.045–0.014	(*150*)
	DLST	Dihydrolipoamide S-succinyltransferase (E2 component of 2-oxo-glutarate complex	1	−0.95	↓	10	0.003	(*151*)
**Miscellaneous**								
**Neuronal outgrowth**	NCAM1	Neural cell adhesion molecule-1	1	−0.27	↓	27	0.027	(*123–125*)
	NFASC	Neurofascin	2	−0.21–(−0.27)	↓	36	0.037–0.017	(*126*)
	DCLK1	Serine/threonine-protein kinase	1	0.67	↑	19	0.027	(*152*)
	NRCAM	Neuronal cell adhesion molecule	1	0.69	↑	31	0.049	(*57*)
**Regulation of nucleic acid metabolism**	HNRNPA2B1	Heterogeneous nuclear ribonucleoprotein A2/B1	1	−0.24	↓	16	0.033	(*128*)
	CMPK1	UMP-CMP kinase	1	0.51	↑	10	0.34	(*129*)
**Immune response**	IGHG2	Ig gamma-2 chain C region	1	0.85	↑	8	0.032	(*62*)
	IGHG1	Ig gamma-1 chain C region	1	0.77	↑	14	0.031	(*62, 63*)
**Protein metabolism**	CTSD	Cathepsin D	3	−0.51–(−0.93)	↓	14	0.05–0.0077	(*132, 133*)
	DDAH1	N(G)-dimethylarginine dimethylaminohydrolase	2	−0.21–(−0.15)	↓	22	0.0058–0.0023	(*69, 70*)
	HSPA1A	Heat shock 70 kDa protein 1A/1B	1	0.36	↑	28	0.025	(*153, 154*)
	PSMA1	Proteasome subunit alpha type-1	1	0.40	↑	10	0.0093	(*155*)
	HSPE1	10kDa heat shock protein	1	−0.37	↓	7	0.015	(*156*)
**Amine metabolism**	MAOB	Amine oxidase B	1	−0.88	↓	18	0.027	(*157*)
**Lysosomal and peroxisomal degradation**	PSAP	Prosaposin	4	−0.97–(−1.20)	↓	13	0.018–0.00017	(*71–73*)

Significantly up- or downregulated proteins in biopsies from patients with diffuse axonal injury (DAI) in comparison to idiopathic normal pressure hydrocephalus (iNPH).

IP = Number of identified peptides, ↑↓ = Increased or decreased, * = Reference provided in supplementary material.

).

The analysis identified 20 proteins with significantly increased or decreased expression in DAI both when compared to focal TBI and when compared to iNPH. Among these, 5 proteins were involved in neurodegeneration or cytoskeletal function (MAPT, MAP6, DMTN, CRMP1, MAP1A). Details of overlap of differentially expressed proteins are shown in Suppl. Table 2 and Fig. 1D.

### Proteome differences between focal TBI and iNPH patients (study B)

LTQ-FT MS and LTQ-Orbitrap MS analysis using LF and DML proteomics identified a total of 1844 unique proteins. Of these, 29 proteins were significantly increased (n = 20) or decreased (n = 9) in focal TBI when compared to iNPH (Table 6). Of the significantly up- or downregulated proteins 10% (n = 3, increased n = 0, decreased n = 3) are involved in neurodegeneration or cytoskeletal function, 14% (n = 4, increased n = 2, decreased n = 2) in cell death or survival functions, 21% (n = 6, increased n = 6, decreased n = 0) cell signaling, transport or repair pathways, 3% (n = 1, increased n = 1, decreased n = 0) in oxidation/reduction pathways and 14% (n = 4, increased n = 2, decreased n = 2) in energy metabolism. Of the remaining 38% (n = 11, increased n = 9, decreased n = 2), identified proteins were involved in neurite outgrowth, protein metabolism, regulation of nucleic acid and immune response (Fig. 1C

**Table 6:** Mass spectrometry analysis of brain tissue biopsies from structurally normal-appearing frontal cortex-*STUDY B*.

Biological process	Gene name	Protein name	Number of platforms	Ratio	↑↓	IP	P-value	Reference*
**Neurodegeneration/Cytoskeleton**	NEFM	Neurofilament medium polypeptide	2	−0.48–(−0.52)	↓	54	0.029	(*158, 159*)
	ARPC1A	Actin-related protein 2/3 complex subunit 1A	1	−0.12	↓	14	0.039	(*160*)
	NEFH	Neurofilament heavy polypeptide	1	−0.93	↓	31	0.00098	(*138, 139*)
**Cell death/survival**	RANBP1	Ran-specific GTPase-activating protein	1	−0.61	↓	5	0.0081	(*161, 162*)
	PRNP	Major prion protein	1	0.24	↑	5	0.022	(*163–165*)
	YWHAE	14-3-3 protein epsilon	1	0.11	↑	26	0.037	(*19–21*)
	UBC	Ubiquitin-60S ribosomal protein	1	−0.31	↓	5	0.041	(*166*)
**ROS/RNS**	PRDX3	Thioredoxin-dependent peroxide reductase	1	0.092	↑	10	0.023	(*104*)
**Transport/Signaling/Repair**	RAB5B	Ras-related protein Rab-5B	1	0.077	↑	7	0.018	(*167*)
	SPARCL1	SPARC-like protein 1	1	0.60	↑	7	0.039	(*168, 169*)
	GDI1	Rab GDP dissociation inhibitor alpha	1	0.28	↑	37	0.044	(*170*)
	RAC	Ras-related C3 botulinum toxin substrate	1	0.27	↑	9	0.021	(*171, 172*)
	FKBP1A	Peptidyl-prolyl cis-trans isomerase	1	0.24	↑	10	0.027	(*173*)
	CNTNAP1	Contactin-associated protein 1	1	0.36	↑	35	0.030	(*118, 119*)
**Energy**	ALDH2	Aldehyde dehydrogenase	1	0.098	↑	26	0.048	(*148, 149*)
	LDHA	L-lactate dehydrogenas A chain	1	0.12	↑	22	0.0055	(*56*)
	DLAT	Dihydrolipoamide S-acetyltransferase	1	−1.26	↓	14	0.042	(*174*)
	CS	Citrate synthase	1	−0.77	↓	18	0.017	(*175*)
**Miscellaneous**								
**Neuronal outgrowth**	NDRG2	Protein NDRG2	1	−0.32	↓	15	0.035	(*176*)
**Regulation of nucleic acid metabolism**	EEF1A2	Elongation factor 1-alpha 2	1	−0.55	↓	20	0.048	(*177*)
	EEF1B2	Elongation factor 1-beta	1	0.098	↑	6	0.020	(*178*)
**Immune response**	SERPINA1	Alpha-1 antitrypsin	1	2.42	↑	23	0.029	(*179, 180*)
	BSG	Basigin	1	0.49	↑	6	0.039	(*181*)
**Protein metabolism**	CTSD	Cathepsin D	1	0.32	↑	14	0.034	(*132, 133*)
	PDIA3	Protein disulfide-isomerase A3	1	0.069	↑	6	0.028	(*182*)
	HSPA5	78 kDA glucose-regulated protein	1	0.33	↑	20	0.048	(*183, 184*)
	GLUD	Glutamate dehydrogenase	1	0.23	↑	31	0.022	(*185, 186*)
	ALDH6A1	Methylmalonate-semialdehyde dehydrogenase	1	0.30	↑	25	0.0012	(*187*)
	A2M	Alpha-2-macroglobulin	1	1.057	↑	50	0.035	(*188, 189*)

Significantly up- or downregulated proteins in biopsies from patients with focal traumatic brain injury (TBI) in comparison to idiopathic normal pressure hydrocephalus (iNPH).

IP = Number of identified peptides, ↑↓ = Increased or decreased, * = Reference provided in supplementary material.

).

### Pathway analysis

A pathway analysis (using Ingenuity Pathway Analysis, Ingenuity Systems, Qiagen) based on the significantly regulated proteins in *Study B* suggests that the top canonical pathways involved in TBI include oxidative phosphorylation, calcium signaling, mitochondrial dysfunction and phagosome maturation (data not shown).

### Western blot and Aβ40 and Aβ42 ELISA analysis

**Figure 2:**
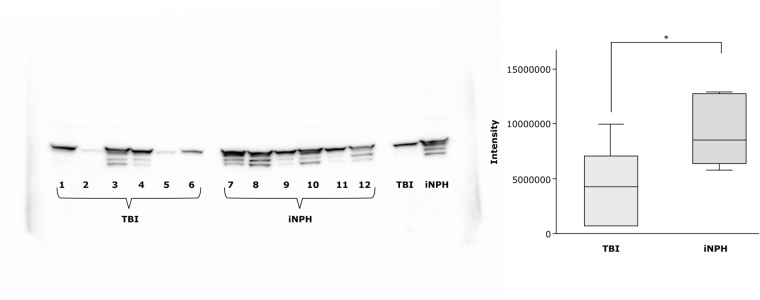
Validation of Glial fibrillary acidic protein (GFAP) expression in *Study A* by Western blot analysis in traumatic brain injury (TBI) vs idiopathic normal pressure hydrocephalus (iNPH) samples. The image shows individual samples from TBI patients (1–6), from iNPH patients (7–12) and pooled samples of TBI and iNPH patients respectively. Similar to the results from the MS-proteomic analyses, the level of GFAP was significantly (*) decreased in TBI as compared to iNPH (*p* = 0.04).

Western blot analysis on glial fibrillary acidic protein (GFAP) from tissue extract were performed to validate the results from *Study A*. The Western blot showed a robust primary GFAP band, in addition to some lower, non-specific molecular bands presumably caused by excess antibody, image exposure time or rapid substrate consumption. The Western blot analysis revealed a significant decrease of GFAP expression in TBI *(p* = 0.04, Fig. 2), similar to the findings in *Study A*.

Amyloid-β (Aβ) is a key contributor in Alzheimer’s disease pathology and linked to axonal injury^14^. However, it is inadequately detected with MS-based proteomics^15^ and therefore we used a sandwich enzyme-linked immunosorbent assay (ELISA) to measure monomeric Aβ peptides in the tissue extracts. The Aβ ELISA analysis revealed no difference in levels of neither Aβ40 nor Aβ42 between patients with DAI (median Aβ40 35 pM, range 30–61 pM and Aβ42 6 pM range 3–11 pM), focal TBI (median Aβ40 50 pM, range 26–118 pM and Aβ42 15 pM range 1–250 pM) and iNPH (median Aβ40 47 pM, range 31–94 pM and Aβ42 5 pM range 3–6752 pM) (Kruskal-Wallis test, *p* = 0.65 and *p* = 0.68 respectively).

## Discussion

In the present report, the proteome profile in structurally normal-appearing cortical tissue of severe TBI patients compared to cortical tissue from patients with iNPH was analyzed using quantitative MS-based proteomics. Fundamental proteome alterations in TBI patients were observed, which were more pronounced in TBI patients with widespread axonal injury, suggesting that this injury type rapidly induces cascades linked to secondary injury pathways which may be related to the risk of developing neurodegenerative disorders.

This study is the first to evaluate tissue biopsies from TBI patients, obtained at the location of ICP monitoring, at a similar location in each patient, and at a distance from any radiologically visible brain injury. Although proteome analysis in TBI has been performed on brain tissue from surgically resected contusions and *post-mortem* brains from patients that succumbed to TBI^16^, the unique cortical samples used in the present study provide insight into global cellular alterations in uninjured brain regions that occur in human TBI. The biopsies were obtained using a minimally traumatizing technique established at our department as a routine procedure for iNPH, safely performed > 400 times during the last years in conjunction with VP shunt placement^17^. In this TBI cohort, one patient had a small hematoma without clinical significance in the area of the brain biopsy and ICP monitoring, in line with the hemorrhage risk from ICP monitoring^18^. The small size of the tissue biopsy pose a challenge for sample preparation. To prevent unspecific loss in proteins by sticking to the surfaces, a combination of homogenization techniques were employed in the presence of detergent based lysis buffer. The brain tissue protein concentrations measured using DC protein assay kit were 200–237 µg, proving the efficiency of the techniques.

We found proteome differences between DAI and focal TBI, which may have important implications for individualized therapies in TBI patients and future development of pharmacological treatments. Previous pharmacological studies have universally failed to provide a clinical benefit in human TBI^12^ where the heterogeneity of TBI is a recognized and major obstacle^13^. Current clinical and radiological classification may be insensitive to the complex biological cascades being markedly different in the different TBI subtypes^1^. In TBI patients, 45 proteins were altered when compared to iNPH patients. More importantly, 20 proteins had altered expression in DAI compared to both iNPH and focal TBI patients. This suggests that DAI causes global alterations in cortical tissue, to a larger degree than focal lesions.

TBI is today an accepted major risk factor for the development of neurodegenerative disease later in life^14,19–21^. In particular, aggregations of Aβ and tau, the hallmark pathology observed in Alzheimer’s disease (AD) patients, have suggested a link between DAI and the subsequent development of neurodegenerative disease^8,10,22^. Histologically, β-amyloid precursor protein (βAPP) accumulates in injured axons^10^ and when cleaved by β- and γ-secretases, β-amyloid (Aβ) peptides are generated^23^. Insoluble Aβ aggregates are found within hours after the injury in approximately 30% of severe TBI cases^24,25^ and also in injured axons^26^. Amyloid-β species are difficult to detect using conventional mass spectrometry due to their hydrophobicity, high mass (>4 kDa), and low abundance^15^. Therefore, we analyzed the levels of aggregation prone Aβ species using highly-sensitive ELISA. However, the Aβ levels were similar in TBI and iNPH as well as between the TBI subtypes. This may be attributed to the use of iNPH patients as study controls since Aβ pathology may exist in iNPH^27^. To date, the cortical Aβ levels of healthy individuals are unknown.

In this study, a considerable number of proteins involved in cytoskeletal function were altered in TBI and, particularly, in DAI. Of these, we observed increased expression of tau in DAI when compared to focal TBI and to iNPH. Tau is a microtubule-associated protein and is an important structural element in the axonal cytoskeleton. In the normal brain, tau is phosphorylated to regulate its biological activity. However, there is an association of excessive phosphorylation of tau (i.e hyperphosphorylation) with a number of neurodegenerative diseases^28^. In AD a common feature is neurofibrillary tangles (NFTs) composed by HPtau aggregates^29^. Tau was elevated both in CSF and in interstitial brain tissue monitored by microdialysis in severe TBI patients^30–32^. Additionally, abundant and widely distributed NFTs were found in 46% of patients who died long after a single moderate-severe TBI^33^. However, tau in the form of NFTs has not been observed in the early post-injury months in TBI, although evidence from animal models and *post-mortem* brains from young athletes dying shortly after sustaining a concussion suggests that HPtau may develop rapidly after trauma^34,35^. Here, increased tau expression in structurally normal-appearing brain tissue early following widespread axonal injury in humans was observed. Analysis of temporal tau patterns were not possible and whether the cortical tau expression in DAI patients results in tau aggregates over time remains to be elucidated. Importantly, tau alterations did not stand out alone, and pronounced alterations were detected in a number of additional proteins. The importance of tau alterations relative to these other proteins in the pathophysiology of DAI cannot be established using the present methods.

Eleven additional proteins involved in cytoskeletal function had altered expression in DAI compared to focal TBI, including Fascin, γ-adducin and glial fibrillary acidic protein (GFAP). Fascin is an actin-binding protein, important for the formation, maintenance, and stability of cellular structures^36^ and adducins regulate synaptic remodeling and control synaptic growth and disassembly during plasticity^37^. Although their role in TBI is unknown, to date, we observed decreased γ-adducin expression in DAI, which may reflect deficits in synaptic stability caused by TBI. Glial fibrillary acidic protein (GFAP), expressed by several cell types including CNS astrocytes, is increased in TBI^38,39^. We found decreased expression of GFAP in TBI compared to iNPH tissue (*Study A*). In *post-mortem* tissue from human TBI, the number of GFAP-positive astrocytes decreased within the first 24 hours, then followed by an increased number indicating reactive gliosis formation^40^. Recently, GFAP kinetics were investigated in astrocytes from a human trauma culture model and in CSF from TBI patients. Release of GFAP and its small degradation products occurred in a delayed fashion and preceded cell death of wounded astrocytes^41^. Additionally, GFAP mRNA increases with age in human brains^42^. In the TBI cohort in *Study A*, more pronounced GFAP increases were noted in older patients or with biopsy obtained >24 hrs post-injury. Nevertheless, we were able to detect an increased GFAP expression in focal TBI when compared to DAI, consistent with the results from serum^39^. These results argue that GFAP levels may vary due to injury type, age of patient and time post-injury.

Notably, no elevations of neurofilaments (NFs) in the brain tissue of TBI patients were observed. In fact, neurofilament heavy peptide (NEFH) was decreased in DAI when compared to iNPH. High levels of neurofilaments, composed of neuron-specific intermediate filaments, were found in ventricular and lumbar CSF as well as in serum^43,44^ following TBI. It is plausible that following axonal injury, NFs may leak into the CSF space and serum, despite unaltered expression in the cortical tissue.

Additional potential TBI biomarkers that were found with altering expression in TBI were neuron-specific enolase (NSE), Neurogranin (NRGN), Cathepsin D (CTSD) and fatty acid-binding protein, heart (FABP3). NSE is localized in the cytoplasm of neurons and is upregulated and associated with poor outcome in DAI^45^. Similar to our present data, serum NSE had different temporal patterns in DAI compared to focal TBI^46^. Neurogranin (NRGN), a postsynaptic protein involved in memory consolidation, predicts cognitive deterioration in prodromal Alzheimer’s disease^47^, and fatty acid-binding protein, heart (FABP3), involved in fatty acid metabolism and lipid transport, is a biomarker candidate for AD, subarachnoid hemorrhage and TBI^48–50^, where the levels in serum can predict mortality^48^. In our study NSE, NRGN and FABP3 were increased. In contrast, the lysosomal protease CTSD decreased in DAI, is linked to defective degradation of Aβ^51^ as well as α-synuclein toxicity in Parkinson disease (PD)^52^. These data enforce the notion that DAI triggers multiple injury cascades that cannot be visualized with currently existing methods, and may reflect an increased vulnerability to long-term neurodegeneration.

The brain is highly sensitive to free radicals and an overproduction of reactive oxygen/nitrogen species (ROS/RNS) is a key secondary injury factor in TBI. The resulting oxidative stress causes damage to cellular membranes and organelles^53^. In our study, we found mainly decreased expression of antioxidant proteins by TBI. Perioxiredoxin-6 (PRDX6) is a major antioxidant enzyme found primarily in astrocytes, increased in AD brains where it may neutralize ROS in the vicinity of Aβ aggregates, and was found oxidized and inactivated in the CSF of severe TBI patients^54^. Consistent with these findings, we detected decreased PRDX6 expression in TBI brain tissue, and particularly in DAI. Protein DJ-1 (PARK7), another antioxidant protein with neuroprotective properties, was also decreased by TBI. Decreased expression of antioxidants may increase the susceptibility to ROS/RNS-mediated secondary brain injury following TBI.

Traumatic axonal injury has consistently been reported to activate microglia and inflammatory pathways, both in animal models as well as in human TBI^22,55,56^, and elevations of pro-inflammatory cytokines and chemokines are observed in serum, CSF and in brain interstitial fluid^56^. In human brain tissue, microglial activation and elevated cytokines are observed in surgically resected contusions^16,57^. Contradictory to previous studies, the measured inflammatory responses induces by TBI in the present study were modest. This could possibly be attributed to the use of structurally normal-appearing cortical tissue at an early stage post-injury. In the acute phase post-injury, inflammation is limited and mainly intravascular, while parenchymal inflammation takes longer to develop^55,58^. In *post-mortem* tissue from patients that succumbed to TBI, reactive astrocytes and microglia were observed in the hemisphere contralateral to the brain injury in patients with longer survival times post-injury, but not in patients dying at the scene of injury. Moreover the lack of blood-brain-barrier compromise in the biopsy tissue may impede recruitment of immune cells^55,58^, further reducing the inflammatory changes measurable in the sampled biopsies. Additionally, a pre-existing inflammatory process may be present in iNPH patients^59^ possibly masking a TBI-related increase of inflammatory mediators.

This study is limited by the small number of patients and the size of the cortical brain tissue sample, which only allows a small brain area to be analyzed. Proteomic analysis was performed on CSF of TBI patients, and suggested to enable a global estimation of ongoing cellular alterations in the injured brain^60,61^. Nonetheless, CSF analysis cannot detect cellular mechanisms altered by TBI if the proteins are not secreted, and may not reflect on-going changes within neuronal and glial tissue. Additionally, since neurologically normal individuals are not available as controls for obvious ethical reasons, we used iNPH patients as control subjects. However, iNPH carries some similarities to AD and neurodegenerative pathology where insoluble Aβ aggregates and tau accumulations are frequent findings^17^. Furthermore, DAI affects mainly patients of younger age, and in our present study DAI patients were younger than the focal TBI and the iNPH patients. This may cause age-related alterations in protein expression partly explaining some findings in this report^62^. Nonetheless, we observed protein alterations linked to neurodegeneration in the TBI cohort and particularly in DAI patients despite their younger age. Since alteration in iNPH patients due to the disease and/or increasing age plausibly attenuated some TBI-induced alterations, the cellular alterations initiated by TBI may be even more profound than suggested by our present results.

The strengths of this report include the use of structurally normal-appearing cortical tissue. Although patients with focal TBI were not evaluated with MRI we cannot exclude that some traumatic axonal injury existed also in those patients. However, DAI is mainly a clinical diagnosis and we perform MRI on clinical and CT criteria^63^. It is unlikely that extensive axonal injury existed in the focal TBI cohort, and the probability of brain lesions not detected by CT in the region of brain biopsy sampling is small. Further, the fresh brain tissue analyzed in this study is more advantageous than brain tissue samples from autopsy studies, since it eliminates tissue changes caused by prolonged *post-mortem* time.

In this study, the first to carry out and analyse brain tissue biopsies of structurally normal-appearing cortex of severe TBI patients, we provide compelling evidence of alterations in multiple cellular pathways in brain regions remote from radiologically evident brain injury. Additionally, we show that DAI and focal TBI subtypes are fundamentally distinct on the molecular level. These findings have relevance for targeted therapy development, and may increase knowledge of the cellular mechanisms underlying neurodegenerative progression following TBI.

## Methods

The Regional Research Ethics Committee at Uppsala University granted permission for all included studies. Written informed consent was obtained from the TBI patient’s closest relative and from each iNPH patient and all research was conducted in accordance with the ethical standards given in the Helsinki Declaration of 1975, as revised in 2008.

### Patients

Patients with severe TBI (n = 16), defined as post resuscitation Glasgow Coma Scale (GCS) score ≤ 8, were conveniently recruited. Criteria for inclusion were age >16 years and depressed level of consciousness with clinical indication for mechanical ventilation and intracranial pressure (ICP) monitoring. Exclusion criteria were previous neurological disorder, known coagulopathy or inability to locate next of kin for informed consent. Detailed demographic and clinical characteristics are shown in Table 1. Patients were endotracheally ventilated and sedated using a combination of intermittent intravenous (i.v) morphine and continuous i.v. propofol infusion. An ICP monitoring device (Codman microsensor ICP express intraparenchymal monitor with a 1.2 mm external diameter (n = 8; Codman Neuro, USA) or an external ventricular drainage (EVD) with a 2.5 mm external diameter (n = 2; Smith Medical, Germany) or both (n = 6) was inserted for continuous measurements of ICP and cerebral perfusion pressure (CPP). Biopsies were taken in structurally normal-appearing brain tissue, assessed on computed tomography (CT), and the relation of the biopsy area to a focal brain injury, if present, is provided in Table 1. Patients with TBI were treated using an ICP- and CPP-guided standard protocol including mild hyperventilation (PaCO_2_ 30–35 mm Hg; 4.0–4.5 kPa), 30° elevated head of bed, volume expansion to normovolemia and a central venous pressure of 0–5 mm Hg^64^. Aim of treatment was to keep ICP at <20 mm Hg and CPP at >60 mm Hg and ICP elevations not controlled by standard therapy or CSF drainage were treated with a propofol- or pentobarbital induced coma or/and a decompressive craniectomy.

### Imaging

An initial admission CT scan was performed and repeat CT scans were liberally obtained. The worst CT was scored using the Marshall classification^65,66^. Patients with suspected DAI from clinical and CT criteria were subsequently evaluated with MRI within 1day – 8 weeks post-injury. The MRI protocol included diffusion weighted (DWI) and susceptibility weighted (SWI) imaging^63^. A postoperative CT or MRI (mean 1day postoperatively, range 0–3 days) was evaluated for the exact placement of the ICP monitor and for the exclusion of hemorrhages related to the cortical biopsy or placement of ICP monitor. In one patient, postoperative radiological controls were not obtained due to transfer to another hospital.

### iNPH patients

Patients with iNPH were preoperatively evaluated with CT or/and MRI to exclude other neurological conditions. A mini-mental state examination (MMSE) as well as preoperative lumbar CSF samples were obtained. Levels of Aβ1-42, total tau, hyperphosphorylated tau (HPtau) in CSF were assessed using commercial ELISA kits following the manufacturer’s protocol (Table 2). A postoperative CT scan (mean 42, range 5–72 days postoperatively) was obtained in all patients.

### Tissue collection and handling

Brain biopsies were taken in conjunction with the insertion of an ICP monitoring device in the same corticotomy. The corticotomy was performed with a sharp syringe to avoid thermal injury to brain tissue. Biopsy needles with 14 gauge (2.11 mm) diameter and an 8 mm side cutting window (Elekta Instrument AB, Innsbruck, Austria) were used and the biopsies thus included both cortical and subcortical brain tissue. Bipolar diathermy was used in the biopsy area to assure hemostasis before placement of the ICP monitor. The relation in size between the biopsy and the EVD is shown in Fig. 1A. In TBI patients, only one biopsy was obtained as mandated by the ethics committee and therefore immunohistochemistry could not be performed. Similarly, 11 brain biopsies were taken from patients with iNPH during the insertion of a ventriculoperitoneal (VP) shunt according to clinical routine procedures in our neurosurgical department^17^. All biopsies from TBI and iNPH patients were put in pre-labeled 1.5 mL Eppendorf tubes and stored in a −80 °C freezer until analyzed. Brain tissue from iNPH patients was also immediately placed in 4% formaldehyde (HistoLab Products AB, Gothenburg, Sweden) during surgery. The tissue was sent directly to the neuropathological department if surgery was performed during daytime. In on-call situations the samples were stored at 4 °C prior to transfer to the neuropathological department. The samples were fixed in 4% formaldehyde for 24 hours, paraffin embedded using Histovax® (HistoLab Products AB) and processed by hardware Tissue tek VIP (Sakura, CA, USA). Six μm microtome sections were cut using Thermo Scientific Microm HM355 S (Cellab Nordica AB, Sollentuna, Sweden) and placed on SuperFrost® plus slides (Menzel-Gläser). Immunohistochemistry on iNPH patients was performed as part of the clinical routine^17^. In brief, immunohistochemistry using antibodies to Aβ (6F/3D, M0872; dilution 1:100, pretreatment with 80% formic acid for 1 hour; Dako, Glostrup, Denmark) and HPtau protein (AT8, MN1020; dilution 1:500; Thermo Scientific, Waltham, MA) was performed. Dako Autostainer plus was implemented and Dako EnVision FLEX detection system was used for visualization of staining results.

### Chemicals and reagents

Acetonitrile (ACN), acetic acid (HAc), formic acid (FA), sodium chloride (NaCl), protease inhibitor cocktail, trifluoroacetic acid, n-octyl-β-D-glucopyranoside (OG), triethyl ammonium bicarbonate (TEAB), formaldehyde CH_2_O (37% (vol/vol)), iodoacetamide (IAA), urea, and dithiothreitol (DTT) were purchased from Sigma Aldrich (St. Louis, MO, USA). Formaldehyde (^13^CD_2_O) (20% (vol/vol), 99% ^13^C, 98% D) and sodium cyanoborodeuteride (NaBD_3_CN) (96% D) were purchased from Isotec (Miamisburg, OH). Sodium cyanoborohydride (NaBH_3_CN) was obtained from Fluka (Buchs, Switzerland). Trypsin/Lys-C mixture (MS grade; Promega, Mannheim, Germany) were used. Ultrapure water was prepared by Milli-Q water purification system (Millipore, Bedford, MA, USA).

### Protein extraction

The brain biopsy samples (10 mg) were homogenized for 60 seconds in a blender (POLYTRON PT 1200, Kinematica) with 0.5 mL of lysis buffer (10 mM Tris-HCl pH 7.4, 0.15 M NaCl, and PBS containing 1% OG) according to Musunuri *et al*.^67^. The total protein concentration in the supernatant was determined using the DC Protein Assay Kit (BioRad Laboratories, Hercules, USA). The DC assay was carried out according to the manufacturer’s instructions using 96-well microtiter plate reader model 680 (BioRad Laboratories).

### Delipidation and protein precipitation

Aliquots (200 μL) of the protein extracts were delipidated with 1.4 mL of ice-cold tri-n-butylphosphate: acetone: methanol mixture (1:12:1) according to Wetterhall *et al*.^68^.

### On-filter tryptic digestion of proteins

Delipidated protein pellets were re-dissolved in 200 μL of digestion buffer (8 M urea, 1% OG in 50% ACN). 100 μL of 100 mM TEAB was added to the protein aliquots to maintain pH at 7.8. Aliquots corresponding to 50 μg of proteins were taken for digestion. An on-filter digestion protocol developed previously^67^ was used for tryptic digestion of the samples using 3 kDa centrifugal filters (Millipore, Tullagreen, Ireland). The dried tryptic peptide mixtures were reconstituted 125 μL of 0.1% TFA and 50 μL of each sample containing ~20 µg of proteins were vacuum centrifuged to dryness prior to stable-isotope dimethyl labeling. The rest of the volume (75 μL) of the samples was used for label free nanoLC-MS/MS approach.

### Stable-isotope dimethyl labeling

Dimethyl labeling was performed according to previously published method^69^. In *Study A*, 20 µg of peptide mixture from TBI and iNPH samples were reconstituted in 100 μL of 100 mM TEAB, mixed with 4 μL of CD_2_O (4%, v/v) and 4 μL CH_2_O (4%, v/v) respectively. After brief vortexing, 4 μL of freshly prepared 0.6 M NaBH_3_CN was added to both TBI and iNPH samples. In *Study B*, the tryptic peptide mixtures containing (~20 µg) digested proteins from DAI, focal TBI, and iNPH brain samples were reconstituted in 100 μL of 100 mM TEAB, mixed with 4 μL of CD_2_O (4%, v/v), ^13^CD_2_O (4%, v/v), and CH_2_O (4%, v/v) respectively. After brief vortexing, 4 μL of freshly prepared 0.6 M NaBH_3_CN was added to iNPH and DAI samples, while 4 μL of 0.6 M NaBD_3_CN was added to focal TBI samples. The mixtures were incubated for 1 h at room temperature while shaking. The reaction was terminated by adding of 16 μL of ammonia (1%, v/v), and then 8 μL of FA (5%, v/v) was added to consume the excess of labeling reagents. After that, the labeled samples from *Study A*, were mixed in a 1:1 ratio (duplex analysis), whereas samples from *Study B* were mixed in a 1:1:1 ratio (triplex analysis, DAI: focal TBI: iNPH, 5 triplexes in total). The mixed samples were desalted on Isolute C18 solid phase extraction columns (1 mL, 50 mg capacity, Biotage, Uppsala, Sweden) as described in Musunuri *et al*.^67^. After desalting, the eluate was vacuum centrifuged to dryness and re-dissolved in 0.1% TFA to a concentration of 0.4 μg/μL prior to nano-LC-MS/MS.

### NanoLC-MS/MS for protein identification

Nano-LC-MS/MS experiments were performed using 7 T hybrid LTQ-FT mass spectrometer (ThermoFisher Scientific, Bremen, Germany) (both *Study A* and *B*) and LTQ-Orbitrap mass spectrometer (ThermoFisher Scientific) (*Study B* only).

### LTQ-FT MS

The nanoLC-MS/MS experiments were performed according to previously published protocol^67^ using a 7 T hybrid LTQ FT mass spectrometer (ThermoFisher Scientific, Bremen, Germany) fitted with a nano-electrospray ionization (ESI) ion source. On-line nanoLC separations were performed using an Agilent 1100 nanoflow system (Agilent Technologies, Waldbronn, Germany).

### LTQ-Orbitrap MS

EASY-nLC II (ThermoFischer Scientific) is used to perform on-line nanoLC separations. The LC setup was connected to an LTQ Orbitrap Velos Pro mass spectrometer (ThermoFischer Scientific, Bremen, Germany) equipped with a nano flex ion source (Proxeon Biosystems). Peptide mixtures were separated using an EASY-Column, 10 cm, inner diameter 75 μm, 3 μm, C18-A2 (Thermo Scientific) according to previously published protocol^70^.

### Western blot analysis

To confirm the protein changes detected by MS-proteomic analyses, western blot detection of glial fibrillary acidic protein (GFAP) was performed. Briefly, 25 µg of proteins from each brain specimen were resolved by SDS-PAGE on 4–12% Bis-Tris Criterion XT Precast Gel (BioRad Laboratories, Solna, Sweden), according to the manufacturer’s instructions. The separated proteins were transferred onto nitrocellulose membrane (Amersham Biosciences GE, Little Chalfont, UK) and blot was blocked for one hour at room temperature with 5% non-fat dry milk dissolved in Tris buffered saline (50 mM Tris, 0.5% Tween-20, pH 8.0), washed briefly with TBS-T twice prior to incubation with primary antibody in 0.5% fat-free milk in TBS-T overnight at 4 °C under gentle agitation. The primary antibody was diluted as indicated and used for immunoblotting: anti-GFAP (1:2000 dilution; from Abcam, Cambridge, MA). The membranes were washed three times, 10 min, with TBS and then incubated with secondary IgG (rabbit-anti-mouse HRP-conjugated) for 90 min. After washing three times with TBS, immunoreactive bands were detected by enhanced chemiluminescence (ECL) detection kit (Amersham Biosciences GE, Little Chalfont, UK) and imaged with ChemiDoc XRS + (Bio-Rad, Hercules, CA). Equal protein loading was verified by a Ponceau red staining of the membranes. Bands were plotted and quantified from tif-files using ImageJ, classical statistical analysis was then performed with GraphPad PRISM 5 using student’s two-tailed t-test.

### Aβ40 and Aβ42 ELISA analysis

The brain extracts, prepared as described above were denatured by 5 min boiling in 1% SDS to obtain a preparation of monomeric Aβx-40 and Aβx-42, since Aβ aggregates are poorly detected with ELISA^71^. Samples were diluted in ELISA sample buffer and analyzed in duplicates with WAKO Aβ40 and WAKO Aβ42, High Sensitive ELISA kits (Wako Chemicals USA, Inc, Richmond, USA). All samples as well as the Aβ standard had the same final concentration of SDS (0.1%) and lysis buffer (10%). This procedure does not interfere with Aβ detection in these ELISA kits^72^. Analyses were made by a researcher blinded to the clinical information.

### Data analysis and statistics

Acquired raw files were processed by MaxQuant (version 1.4.0.1). Tandem mass spectra were searched with Andromeda against the UniProt human database (release January 2015). The searching settings were set as: maximum 10 ppm and 5 ppm error tolerance for the survey scan and MS/MS analysis respectively; enzyme specificity was trypsin/Lys-C; maximum two missed cleavage sites were allowed; cysteine carbamidomethylation was set as static modification, and Oxidation (M) was set as dynamic modification. For dimethyl labelling additional parameters were added in searches: Dimethyl (K); Dimethyl (N-term); Dimethyl: 2H (4) (K) and Dimethyl: 2H (4) (N-term).

The search criteria for protein identification were set to at least two matching peptides. No proteins were identified and quantified using only one peptide. A maximum false discovery rate (FDR) of 1% for peptides and proteins was selected. Both razor and unique peptides were used for quantification. A decoy sequence database was built by reversing the target sequence database. A list of known contamination was also included in the identification. The protein intensity values were used for further data analysis.

Statistica 12.0 (Stat Soft, Inc. Tulsa, OK) was used for descriptive and analytical statistics. The Kolmogorov-Smirnov test of normality was used to determine the distribution of the variables. Student’s t- test was used for normal distributed data and Mann-Whitney U test for skewed distribution. For comparison of protein intensities, Student’s t- test was performed. Two-tailed *p*-values were used and a *p*-value < *0.05* was considered statistically significant.

## Electronic supplementary material

Supplementary material
