# Supplementary material for: Proteomic differences between focal and diffuse traumatic brain injury in human brain tissue
Source: Sci Rep. 2018 May 1;8:6807. doi: 10.1038/s41598-018-25060-0 (PMC5931620; doi:10.1038/s41598-018-25060-0)
Supplement: Supplementary file 1 — Supplementary material [file 41598_2018_25060_MOESM1_ESM.pdf]

Proteomic differences between focal and diffuse traumatic brain injury in human brain tissue

Sami Abu Hamdeh, MD[1], Ganna Shevchenko PhD[2], Jia Mi PhD[2,3], Sravani Musunuri PhD[2], Jonas Bergquist MD PhD[2, 3†], Niklas Marklund MD PhD[1†*]

[†] Shared senior authorship

[1]Dept of Neuroscience, Neurosurgery, Uppsala University, Uppsala, Sweden

[2]Analytical Chemistry, Department of Chemistry-BMC, Uppsala University, Uppsala, Sweden

[3]Medicine and Pharmacy Research Center, Binzhou Medical University, Yantai, China

[*] Corresponding Author

Niklas Marklund

Professor at the Dept. of Neurosurgery

Uppsala University Hospital

751 85 Uppsala

Sweden

E-mail: niklas.marklund@neuro.uu.se.

Supplementary figure 1.

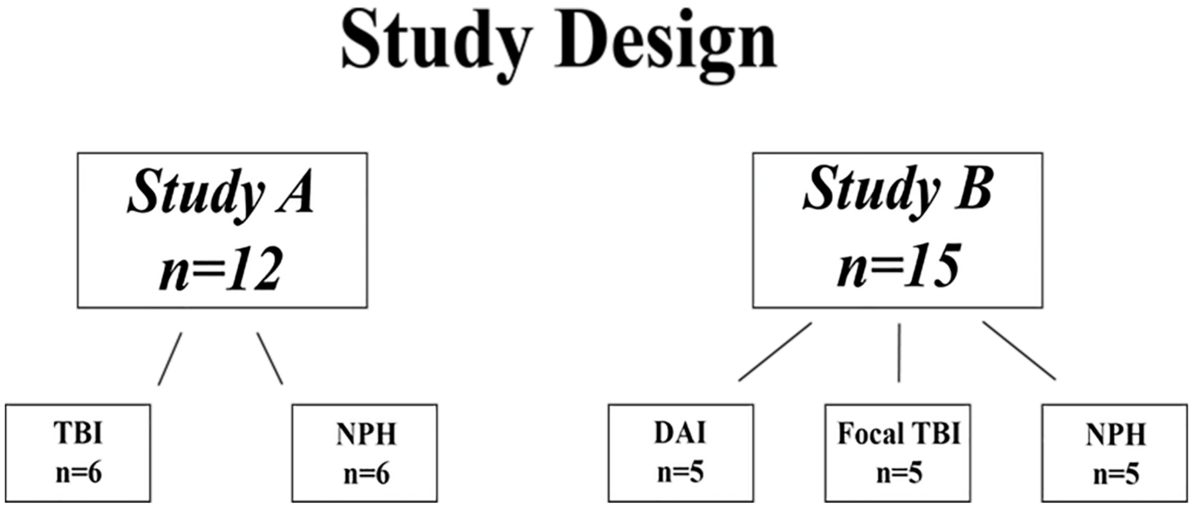

Description of study design. Two separate proteomics analysis were performed. *Study A* included biopsies from a cohort of severe traumatic brain injury (TBI) patients that were compared to biopsies from idiopathic normal pressure hydrocephalus (iNPH). *Study B* included three groups; group A with biopsies from patients with diffuse axonal injury (DAI), group B with biopsies from patients with focal traumatic brain injury and group C with biopsies from patients with idiopathic normal pressure hydrocephalus (iNPH).

Supplementary tables

Suppl. Table 1 Genes with significant differences in expression between groups

STUDY A: TBI VS INPH	STUDY B: DAI VS FOCAL TBI	STUDY B: DAI VS INPH	STUDY B: FOCAL VS INPH
CADM2	MAPT	MAPT	NEFM
TBB2A	MAP6	MAP6	ARPC1A
TBB5	FSCN1	MAPT1	NEFH
SPTA2	DMTN	DMTN	RANBP1
SPTB2	TPM1	CRMP1	PRNP
GFAP	ADD3	MAP1A	YWHAE
MARCS	GFAP	NEFH	UBC
VIME	CRMP1	YWHAB	PRDX3
EPB41L3	MAP1A	YWHAZ	RAB5B
UBE2N	EPB41L3	NPEPPS	SPARCL1
YWHAE	MAP1B	BCAN	GDI1
UBA1	CAPZB	UBB	RAC
CRYAB	YWHAB	CRYM	FKBP1A
MDHC	PPP3R1	PRDX6	CNTNAP1
PRDX6	YWHAQ	GSTO1	ALDH2
PARK7	YWHAE	GGCT	LDHA
PRDX2	CD47	FABP3	DLAT
RAB3A	CDC42	AP2A1	CS
SYPH	CRYM	NRGN	NDRG2
SYN1	PRDX6	SCRN1	EEF1A2
SCRN1	GSTM3	NCALD	EEF1B2
REEP5	PRDX3	ENSA	SERPINA1
4F2	FABP3	PKM	BSG
CNTN1	AP2A1	ALDH2	CTSD
PEBP1	DBNL	TPI1	PDIA3
TRFE	NRGN	DLST	HSPA5
HBA	SCRN1	NCAM1	GLUD
CAH2	VSNL1	NFASC	ALDH6A1
PKM	GNB1	DCLK1	A2M
ATP5J	NCALD	NRCAM	
ALDOC	SEP_11	HNRNPA2B1	
NSE	CLTA	CMPK1	
LDHB	CNTNAP1	IGHG2	
LDHA	PKM	IGHG1	
NRCAM	PYGB	CTSD	
IGSF8	ALDOC	DDAH1	
LSAMP	NSE	HSPA1A	
HPRT	LDHB	PSMA1	
IGHG1	TKT	HSPE1	
IGKC	NCAM1	MAOB	
HS90A	NFASC	PSAP	
ALDH6A1	TNR		
VPP1	HNRNPA2B1		
DDAH1	CMPK1		
PSAP	GDA		
	HIST1H2BN		
	CTSD		
	ALDH6A1		
	CALR		
	PSAP		
	PHYHIP		

TBI = traumatic brain injury, NPH = normal pressure hydrocephalus, DAI = Diffuse axonal injury

Suppl. Table 2. Differentially expressed proteins with overlap between Study A and Study B and between groups in Study B. DAI = diffuse axonal injury, FOCAL = focal traumatic brain injury, iNPH = idiopathic normal pressure hydrocephalus

**OVERLAPPING PROTEINS BETWEEN STUDY A AND STUDY B**				
**STUDY A AND STUDY B (DAI VS FOCAL, DAI VS INPH)**	**STUDY A AND STUDY B (DAI VS FOCAL, FOCAL VS INPH)**	**STUDY A AND STUDY B (DAI VS FOCAL)**	**STUDY A AND STUDY B (DAI VS INPH)**	**STUDY A AND STUDY B (FOCAL VS INPH)**
PRDX6, SCRN1, PKM	YWHAE, ALDH6A1	GFAP, EPB41L3, ALDOC, NSE, LDHB	NRCAM, IGHG1, DDAH1	LDHA
**OVERLAPPING PROTEINS BETWEEN GROUPS IN STUDY B**				
**STUDY B (DAI VS FOCAL, DAI VS INPH, FOCAL VS NPH)**	**STUDY B (DAI VS FOCAL, DAI VS INPH)**	**STUDY B DAI VS NPH, FOCAL VS INPH**	**STUDY B DAI VS FOCAL, FOCAL VS INPH**	
CTSD	MAPT, MAP6, DMTN, CRMP1, MAP1A, YWHAB, CRYM, PRDX6, FABP3, AP2A1, NRGN, SCRN1, NCALD, PKM, NCAM1, NFASC, HNRNPA2B1, CMPK1, PSAP	NEFH, ALDH2	YWHAE, PRDX3, CNTNAP1, ALDH6A1	

Suppl. Table 3. Gene abbreviations

4F2 = 4F2 cell-surface antigen heavy chain	A2M = Alpha-2-macroglobulin
ADD3 = Gamma-adducin	ALDH2 = Aldehyde dehydrogenase
ALDH6A1 = Methylmalonate-semialdehyde dehydrogenase	ALDOC = Fructose-bisphosphate aldolase C
AP2A1 = AP-2 complex subunit alpha-1	ARPC1A = Actin-related protein 2/3 complex subunit 1A
ATP5J = ATP synthase-coupling factor 6	BCAN = Brevican core protein
BSG = Basigin	CADM2 = Cell adhesion molecule 2
CAH2 = Carbonic anhydrase 2	CALR = Calreticulin
CAPZB = F-actin-capping protein subunit beta	CD47 = Leukocyte surface antigen CD47
CDC42 = Cell division control protein 42 homolog	CLTA = Clathrin light chain A
CMPK1 = UMP-CMP kinase	CNTN1 = Contactin-1
CNTNAP1 = Contactin-associated protein 1	CRMP1 = Dihydropyrimidinase-related protein 1
CRYAB = Alpha-crystallin B chain	CRYM = Ketamine reductase
CS = Citrate synthase	CTSD = Cathepsin D
DBNL= Drebin-like protein	DCLK1 = Serine/threonine-protein kinase
DDAH1 = N(G)-dimethylarginine dimethylaminohydrolase	DLAT = Dihydrolipoamide S-acetyltransferase
DLST = Dihydrolipoamide S-succinyltransferase (E2 component of 2-oxo-glutarate complex	DMTN = Dematin
EEF1A2 = Elongation factor 1-alpha 2	EEF1B2 = Elongation factor 1-beta
NSE = Neuron specific enolase	ENSA = Alpha-endosulfine
EPB41L3 = Band 4.1-like protein 3	FABP3 = Fatty acid-binding protein, heart
FKBP1A = Peptidyl-prolyl cis-trans isomerase	FSCN1 = Fascin
GDA = Guanine deaminase	GDI1 = Rab GDP dissociation inhibitor alpha
GFAP = Glial fibrillary acidic protein	GGCT = Gamma-glutamylcyclotransferase
GLUD = Glutamate dehydrogenase	GNB1 = Guanine nucleotide-binding protein beta-1
GSTM3 = Glutathione S-transferase Mu 3	GSTO1 = Glutathione S-transferase omega-1
HBA = Hemoglobin subunit alpha	HIST1H2BN = Histone H2B type 1
HNRNPA2B1 = Heterogeneous nuclear ribonucleoprotein A2/B1	HPRT = Hypoxanthine-guanine phosphoribosyltransferase
HS90A = Heat shock protein HSP 90-alpha	HSPA1A = Heat shock 70 kDa protein 1A/1B
HSPA5 =78 kDA glucose-regulated protein	HSPE1 =10kDa heat shock protein
IGHG1 = Ig gamma-1 chain C region	IGHG2 = Ig gamma-2 chain C region
IGKC = Ig kappa chain C region	IGSF8 = Immunoglobulin superfamily member 8
LDHA = L-lactate dehydrogenas A chain	LDHB = L-lactate dehydrogenase B-chain
LSAMP = Limbic system-associated membrane protein	MAOB = Amine oxidase B
MAP1A = Microtubule-associated protein 1A	MAP1B = Microtubule-associated protein 1B
MAP6 = Microtubule associated protein 6	MAPT = Microtubule- associated protein tau
MAPT1 = Microtubule- associated protein tau	MARCS = Myristoylated alanine-rich C-kinase substrate
MDHC = Malate dehydrogenas, cytoplasmic	NCALD = Neurocalcin-delta
NCAM1 = Neural cell adhesion molecule-1	NDRG2 = Protein NDRG2
NEFH = Neurofilament heavy polypeptide	NEFM = Neurofilament medium polypeptide
NFASC = Neurofascin	NPEPPS = Puromycin-sensitive aminopeptidase
NRCAM = Neuronal cell adhesion molecule	NRGN = Neurogranin
PARK7 = Protein DJ-1	PDIA3 = Protein disulfide-isomerase A3
PEBP1 = Phosphatidylethanolamine-binding protein 1	PHYHIP = Phytanoyl-CoA hydroxylase-interacting protein
PKM = Pyruvate kinase	PPP3R1 = Calcineurin subunit B type 1
PRDX2 = Peroxiredoxin-2	PRDX3 = Thioredoxin-dependent peroxide reductase
PRDX6 = Peroxiredoxin-6	PSAP = Prosaposin

PSMA1 = Proteasome subunit alpha type-1	PYGB = Glycogen phosphorylase, brain form
RAB3A = Ras-related protein Rab-3A	RAB5B = Ras-related protein Rab-5B
RAC = Ras-related C3 botulinum toxin substrate	RANBP1 = Ran-specific GTPase-activating protein
REEP5 = Receptor expression-enhancing protein 5	SCRN1 = Secernin-1
SEP_11 = Septin-11	SERPINA1 = Alpha-1 antitrypsin
SPARCL1 = SPARC-like protein 1	SPTA2 = Spectrin alpha chain, non-erythrocytic 1
SPTB2 = Spectrin beta chain, non-erythrocytic 1	SYN1 = Synapsin-1
SYPH = Synaptophysin	TBB2A = Tubulin beta-2A chain
TBB5 = Tubulin beta chain	TKT = Transketolase
TNR = Tenascin-R	TPI1 = Triosephosphate isomerase
TPM1 = Tropomyosin alpha-1 chain	TRFE = Serotransferrin
UBA1 = Ubiquitin-like modifier-activating enzyme 1	UBB = Ubiquitin B
UBC = Ubiquitin-60S ribosomal protein	UBE2N = Ubiquitin-conjugating enzyme E2 N
VIME = Vimentin	VPP1 = V-type proton ATPase 116 kDa subunit a
VSNL1 = Visinin-like protein 1	YWHAB = 14-3-3 protein beta/alpha
YWHAE = 14-3-3 protein epsilon	YWHAQ = 14-3-3 protein theta
YWHAZ = 14-3-3 protein zeta/delta	

Supplementary references

1. Yang, S., *et al.* Low CADM2 expression predicts high recurrence risk of hepatocellular carcinoma patients after hepatectomy. *Journal of cancer research and clinical oncology* **140**, 109-116 (2014).
2. Cushion, T.D., *et al.* De novo mutations in the beta-tubulin gene TUBB2A cause simplified gyral patterning and infantile-onset epilepsy. *American journal of human genetics* **94**, 634-641 (2014).
3. Leandro-Garcia, L.J., *et al.* Tumoral and tissue-specific expression of the major human beta-tubulin isotypes. *Cytoskeleton (Hoboken, N.J.)* **67**, 214-223 (2010).
4. Breuss, M., *et al.* Mutations in the beta-tubulin gene TUBB5 cause microcephaly with structural brain abnormalities. *Cell reports* **2**, 1554-1562 (2012).
5. Mondello, S., *et al.* alphaII-spectrin breakdown products (SBDPs): diagnosis and outcome in severe traumatic brain injury patients. *J Neurotrauma* **27**, 1203-1213 (2010).
6. Hamdan, F.F., *et al.* Identification of a novel in-frame de novo mutation in SPTAN1 in intellectual disability and pontocerebellar atrophy. *European journal of human genetics : EJHG* **20**, 796-800 (2012).
7. Saitsu, H., *et al.* Dominant-negative mutations in alpha-II spectrin cause West syndrome with severe cerebral hypomyelination, spastic quadriplegia, and developmental delay. *American journal of human genetics* **86**, 881-891 (2010).
8. Smith, S.A., *et al.* Dysfunction in the betaII spectrin-dependent cytoskeleton underlies human arrhythmia. *Circulation* **131**, 695-708 (2015).
9. Kobeissy, F.H., *et al.* Degradation of betaII-Spectrin Protein by Calpain-2 and Caspase-3 Under Neurotoxic and Traumatic Brain Injury Conditions. *Molecular neurobiology* **52**, 696-709 (2015).
10. Chen, Y., Yu, P., Lu, D., Tagle, D.A. & Cai, T. A novel isoform of beta-spectrin II localizes to cerebellar Purkinje-cell bodies and interacts with neurofibromatosis type 2 gene product schwannomin. *Journal of molecular neuroscience : MN* **17**, 59-70 (2001).
11. Okonkwo, D.O., *et al.* GFAP-BDP as an acute diagnostic marker in traumatic brain injury: results from the prospective transforming research and clinical knowledge in traumatic brain injury study. *J Neurotrauma* **30**, 1490-1497 (2013).
12. Pinner, A.L., Haroutunian, V. & Meador-Woodruff, J.H. Alterations of the myristoylated, alanine-rich C kinase substrate (MARCKS) in prefrontal cortex in schizophrenia. *Schizophrenia research* **154**, 36-41 (2014).
13. Jarboe, J.S., *et al.* MARCKS regulates growth and radiation sensitivity and is a novel prognostic factor for glioma. *Clinical cancer research : an official journal of the American Association for Cancer Research* **18**, 3030-3041 (2012).
14. Shirahata, A. & Hibi, K. Serum vimentin methylation as a potential marker for colorectal cancer. *Anticancer research* **34**, 4121-4125 (2014).

15. Levin, E.C., *et al.* Neuronal expression of vimentin in the Alzheimer's disease brain may be part of a generalized dendritic damage-response mechanism. *Brain research* **1298**, 194-207 (2009).
16. Wang, Z., *et al.* Tumor suppressor role of protein 4.1B/DAL-1. *Cellular and molecular life sciences : CMLS* **71**, 4815-4830 (2014).
17. Cheng, J., *et al.* A small-molecule inhibitor of UBE2N induces neuroblastoma cell death via activation of p53 and JNK pathways. *Cell death & disease* **5**, e1079 (2014).
18. Yin, P., *et al.* Aged monkey brains reveal the role of ubiquitin-conjugating enzyme UBE2N in the synaptosomal accumulation of mutant huntingtin. *Human molecular genetics* **24**, 1350-1362 (2015).
19. Steinacker, P., Aitken, A. & Otto, M. 14-3-3 proteins in neurodegeneration. *Seminars in cell & developmental biology* **22**, 696-704 (2011).
20. Krzyzanowska, A., *et al.* Expression of regulatory proteins in choroid plexus changes in early stages of Alzheimer disease. *Journal of neuropathology and experimental neurology* **74**, 359-369 (2015).
21. Shimada, T., Fournier, A.E. & Yamagata, K. Neuroprotective function of 14-3-3 proteins in neurodegeneration. *BioMed research international* **2013**, 564534 (2013).
22. Moudry, P., *et al.* Ubiquitin-activating enzyme UBA1 is required for cellular response to DNA damage. *Cell cycle (Georgetown, Tex.)* **11**, 1573-1582 (2012).
23. Reddy, V.S. & Reddy, G.B. Emerging role for alphaB-crystallin as a therapeutic agent: pros and cons. *Current molecular medicine* **15**, 47-61 (2015).
24. Lo, A.S., *et al.* Developmental regulation and cellular distribution of human cytosolic malate dehydrogenase (MDH1). *Journal of cellular biochemistry* **94**, 763-773 (2005).
25. Manevich, Y., *et al.* Peroxiredoxin VI oxidation in cerebrospinal fluid correlates with traumatic brain injury outcome. *Free radical biology & medicine* **72**, 210-221 (2014).
26. Krapfenbauer, K., Engidawork, E., Cairns, N., Fountoulakis, M. & Lubec, G. Aberrant expression of peroxiredoxin subtypes in neurodegenerative disorders. *Brain research* **967**, 152-160 (2003).
27. Richarme, G., *et al.* Parkinsonism-associated protein DJ-1/Park7 is a major protein deglycase that repairs methylglyoxal- and glyoxal-glycated cysteine, arginine, and lysine residues. *The Journal of biological chemistry* **290**, 1885-1897 (2015).
28. Lin, J.P., *et al.* DJ-1 is activated in medulloblastoma and is associated with cell proliferation and differentiation. *World journal of surgical oncology* **12**, 373 (2014).
29. Zondler, L., *et al.* DJ-1 interactions with alpha-synuclein attenuate aggregation and cellular toxicity in models of Parkinson's disease. *Cell death & disease* **5**, e1350 (2014).
30. Salzano, S., *et al.* Linkage of inflammation and oxidative stress via release of glutathionylated peroxiredoxin-2, which acts as a danger signal. *Proceedings of the National Academy of Sciences of the United States of America* **111**, 12157-12162 (2014).
31. Lu, W., *et al.* Peroxiredoxin 2 is upregulated in colorectal cancer and contributes to colorectal cancer cells' survival by protecting cells from oxidative stress. *Molecular and cellular biochemistry* **387**, 261-270 (2014).
32. Anitei, M., Cowan, A.E., Pfeiffer, S.E. & Bansal, R. Role for Rab3a in oligodendrocyte morphological differentiation. *Journal of neuroscience research* **87**, 342-352 (2009).
33. Martin, S.B., *et al.* Synaptophysin and synaptojanin-1 in Down syndrome are differentially affected by Alzheimer's disease. *Journal of Alzheimer's disease : JAD* **42**, 767-775 (2014).
34. Shen, Y.C., *et al.* Genetic and functional analyses of the gene encoding synaptophysin in schizophrenia. *Schizophrenia research* **137**, 14-19 (2012).
35. Qin, S., Hu, X.Y., Xu, H. & Zhou, J.N. Regional alteration of synapsin I in the hippocampal formation of Alzheimer's disease patients. *Acta neuropathologica* **107**, 209-215 (2004).
36. Garcia, C.C., *et al.* Identification of a mutation in synapsin I, a synaptic vesicle protein, in a family with epilepsy. *Journal of medical genetics* **41**, 183-186 (2004).
37. Martins-de-Souza, D., *et al.* Proteomic analysis identifies dysfunction in cellular transport, energy, and protein metabolism in different brain regions of atypical frontotemporal lobar degeneration. *Journal of proteome research* **11**, 2533-2543 (2012).
38. Miyoshi, N., *et al.* SCRN1 is a novel marker for prognosis in colorectal cancer. *Journal of surgical oncology* **101**, 156-159 (2010).
39. Way, G., Morrice, N., Smythe, C. & O'Sullivan, A.J. Purification and identification of secernin, a novel cytosolic protein that regulates exocytosis in mast cells. *Molecular biology of the cell* **13**, 3344-3354 (2002).

40. Behrens, M., *et al.* Members of RTP and REEP gene families influence functional bitter taste receptor expression. *The Journal of biological chemistry* **281**, 20650-20659 (2006).
41. Kobayashi, K., *et al.* Enhanced tumor growth elicited by L-type amino acid transporter 1 in human malignant glioma cells. *Neurosurgery* **62**, 493-503; discussion 503-494 (2008).
42. Rome, C., Loiseau, H., Arsaut, J., Roullot, V. & Couillaud, F. Diversity of contactin mRNA in human brain tumors. *Molecular carcinogenesis* **45**, 774-785 (2006).
43. Querol, L., *et al.* Antibodies to contactin-1 in chronic inflammatory demyelinating polyneuropathy. *Annals of neurology* **73**, 370-380 (2013).
44. Zhao, J. & Wenzel, S. Interactions of RKIP with inflammatory signaling pathways. *Critical reviews in oncogenesis* **19**, 497-504 (2014).
45. de Arriba Zerpa, G.A., *et al.* Alternative splicing prevents transferrin secretion during differentiation of a human oligodendrocyte cell line. *Journal of neuroscience research* **61**, 388-395 (2000).
46. Kauwe, J.S., *et al.* Suggestive synergy between genetic variants in TF and HFE as risk factors for Alzheimer's disease. *American journal of medical genetics. Part B, Neuropsychiatric genetics : the official publication of the International Society of Psychiatric Genetics* **153b**, 955-959 (2010).
47. Vichinsky, E.P. Alpha thalassemia major--new mutations, intrauterine management, and outcomes. *Hematology. American Society of Hematology. Education Program*, 35-41 (2009).
48. Hu, X., Huang, Z., Liao, Z., He, C. & Fang, X. Low CA II expression is associated with tumor aggressiveness and poor prognosis in gastric cancer patients. *International journal of clinical and experimental pathology* **7**, 6716-6724 (2014).
49. Kwapis, J.L. & Helmstetter, F.J. Does PKM(zeta) maintain memory? *Brain research bulletin* **105**, 36-45 (2014).
50. Glanzman, D.L. PKM and the maintenance of memory. *F1000 biology reports* **5**, 4 (2013).
51. Zhu, H., *et al.* Over-expression of the ATP5J gene correlates with cell migration and 5-fluorouracil sensitivity in colorectal cancer. *PloS one* **8**, e76846 (2013).
52. Sultana, R., *et al.* Redox proteomic analysis of carbonylated brain proteins in mild cognitive impairment and early Alzheimer's disease. *Antioxidants & redox signaling* **12**, 327-336 (2010).
53. Lazarus, R.C., Buonora, J.E., Jacobowitz, D.M. & Mueller, G.P. Protein carbonylation after traumatic brain injury: cell specificity, regional susceptibility, and gender differences. *Free radical biology & medicine* **78**, 89-100 (2015).
54. Yokobori, S., *et al.* Biomarkers for the clinical differential diagnosis in traumatic brain injury--a systematic review. *CNS neuroscience & therapeutics* **19**, 556-565 (2013).
55. Chen, Y., *et al.* Elevation of serum l-lactate dehydrogenase B correlated with the clinical stage of lung cancer. *Lung cancer (Amsterdam, Netherlands)* **54**, 95-102 (2006).
56. Li, J., *et al.* Suppression of lactate dehydrogenase A compromises tumor progression by downregulation of the Warburg effect in glioblastoma. *Neuroreport* **27**, 110-115 (2016).
57. Sakurai, T. The role of NrCAM in neural development and disorders--beyond a simple glue in the brain. *Molecular and cellular neurosciences* **49**, 351-363 (2012).
58. Gordon-Alonso, M., *et al.* EWI-2 association with alpha-actinin regulates T cell immune synapses and HIV viral infection. *Journal of immunology (Baltimore, Md. : 1950)* **189**, 689-700 (2012).
59. Kolesnikova, T.V., *et al.* Glioblastoma inhibition by cell surface immunoglobulin protein EWI-2, in vitro and in vivo. *Neoplasia (New York, N.Y.)* **11**, 77-86, 74p following 86 (2009).
60. Pimenta, A.F., *et al.* The limbic system-associated membrane protein is an Ig superfamily member that mediates selective neuronal growth and axon targeting. *Neuron* **15**, 287-297 (1995).
61. Fu, R., *et al.* Genotype-phenotype correlations in neurogenetics: Lesch-Nyhan disease as a model disorder. *Brain : a journal of neurology* **137**, 1282-1303 (2014).
62. Aurivillius, M., Oymar, K. & Oxelius, V.A. Immunoglobulin heavy G2 chain (IGHG2) gene restriction in the development of severe respiratory syncytial virus infection. *Acta paediatrica (Oslo, Norway : 1992)* **94**, 414-418 (2005).
63. Pan, B., Zheng, S., Liu, C. & Xu, Y. Suppression of IGHG1 gene expression by siRNA leads to growth inhibition and apoptosis induction in human prostate cancer cell. *Molecular biology reports* **40**, 27-33 (2013).
64. Stavnezer-Nordgren, J., Kekish, O. & Zegers, B.J. Molecular defects in a human immunoglobulin kappa chain deficiency. *Science (New York, N.Y.)* **230**, 458-461 (1985).
65. Ou, J.R., Tan, M.S., Xie, A.M., Yu, J.T. & Tan, L. Heat shock protein 90 in Alzheimer's disease. *BioMed research international* **2014**, 796869 (2014).

66. Kedishvili, N.Y., *et al.* CoA-dependent methylmalonate-semialdehyde dehydrogenase, a unique member of the aldehyde dehydrogenase superfamily. cDNA cloning, evolutionary relationships, and tissue distribution. *The Journal of biological chemistry* **267**, 19724-19729 (1992).
67. Norgett, E.E., *et al.* V1 and V0 domains of the human H+-ATPase are linked by an interaction between the G and a subunits. *The Journal of biological chemistry* **282**, 14421-14427 (2007).
68. Capecci, J. & Forgac, M. The function of vacuolar ATPase (V-ATPase) a subunit isoforms in invasiveness of MCF10a and MCF10CA1a human breast cancer cells. *The Journal of biological chemistry* **288**, 32731-32741 (2013).
69. Saratsis, A.M., *et al.* Insights into pediatric diffuse intrinsic pontine glioma through proteomic analysis of cerebrospinal fluid. *Neuro-oncology* **14**, 547-560 (2012).
70. Zhang, P., *et al.* DDAH1 deficiency attenuates endothelial cell cycle progression and angiogenesis. *PloS one* **8**, e79444 (2013).
71. Gieselmann, V., Zlotogora, J., Harris, A., Wenger, D.A. & Morris, C.P. Molecular genetics of metachromatic leukodystrophy. *Human mutation* **4**, 233-242 (1994).
72. Siri, L., *et al.* A novel homozygous splicing mutation in PSAP gene causes metachromatic leukodystrophy in two Moroccan brothers. *Neurogenetics* **15**, 101-106 (2014).
73. Meyer, R.C., Giddens, M.M., Coleman, B.M. & Hall, R.A. The protective role of prosaposin and its receptors in the nervous system. *Brain research* **1585**, 1-12 (2014).
74. Brody, D.L., Benetatos, J., Bennett, R.E., Klemenhagen, K.C. & Mac Donald, C.L. The pathophysiology of repetitive concussive traumatic brain injury in experimental models; new developments and open questions. *Molecular and cellular neurosciences* **66**, 91-98 (2015).
75. Kondo, A., *et al.* Antibody against early driver of neurodegeneration cis P-tau blocks brain injury and tauopathy. *Nature* **523**, 431-436 (2015).
76. Ling, H., Hardy, J. & Zetterberg, H. Neurological consequences of traumatic brain injuries in sports. *Molecular and cellular neurosciences* **66**, 114-122 (2015).
77. Magnoni, S., *et al.* Tau elevations in the brain extracellular space correlate with reduced amyloid-beta levels and predict adverse clinical outcomes after severe traumatic brain injury. *Brain : a journal of neurology* **135**, 1268-1280 (2012).
78. Magnoni, S., *et al.* Quantitative assessments of traumatic axonal injury in human brain: concordance of microdialysis and advanced MRI. *Brain : a journal of neurology* **138**, 2263-2277 (2015).
79. Marklund, N., *et al.* Monitoring of brain interstitial total tau and beta amyloid proteins by microdialysis in patients with traumatic brain injury. *Journal of neurosurgery* **110**, 1227-1237 (2009).
80. Shultz, S.R., *et al.* Sodium selenate reduces hyperphosphorylated tau and improves outcomes after traumatic brain injury. *Brain : a journal of neurology* **138**, 1297-1313 (2015).
81. Tsitsopoulos, P.P. & Marklund, N. Amyloid-beta Peptides and Tau Protein as Biomarkers in Cerebrospinal and Interstitial Fluid Following Traumatic Brain Injury: A Review of Experimental and Clinical Studies. *Frontiers in neurology* **4**, 79 (2013).
82. Benardais, K., *et al.* Loss of STOP protein impairs peripheral olfactory neurogenesis. *PloS one* **5**, e12753 (2010).
83. Volle, J., *et al.* Reduced expression of STOP/MAP6 in mice leads to cognitive deficits. *Schizophrenia bulletin* **39**, 969-978 (2013).
84. Elkhatib, N., *et al.* Fascin plays a role in stress fiber organization and focal adhesion disassembly. *Current biology : CB* **24**, 1492-1499 (2014).
85. Brandt, G.S. & Bailey, S. Dematin, a human erythrocyte cytoskeletal protein, is a substrate for a recombinant FIKK kinase from Plasmodium falciparum. *Molecular and biochemical parasitology* **191**, 20-23 (2013).
86. Rana, A.P., Ruff, P., Maalouf, G.J., Speicher, D.W. & Chishti, A.H. Cloning of human erythroid dematin reveals another member of the villin family. *Proceedings of the National Academy of Sciences of the United States of America* **90**, 6651-6655 (1993).
87. Redwood, C. & Robinson, P. Alpha-tropomyosin mutations in inherited cardiomyopathies. *Journal of muscle research and cell motility* **34**, 285-294 (2013).
88. Kruer, M.C., *et al.* Mutations in gamma adducin are associated with inherited cerebral palsy. *Annals of neurology* **74**, 805-814 (2013).
89. Chang, R.Y., Etheridge, N., Dodd, P.R. & Nouwens, A.S. Targeted quantitative analysis of synaptic proteins in Alzheimer's disease brain. *Neurochemistry international* **75**, 66-75 (2014).

90. Halpain, S. & Dehmelt, L. The MAP1 family of microtubule-associated proteins. *Genome biology* **7**, 224 (2006).
91. Maruyama, K.*, et al.* Beta-actinin is equivalent to Cap Z protein. *The Journal of biological chemistry* **265**, 8712-8715 (1990).
92. Mukaihara, K.*, et al.* Expression of F-actin-capping protein subunit beta, CAPZB, is associated with cell growth and motility in epithelioid sarcoma. *BMC cancer* **16**, 206 (2016).
93. Cruchaga, C.*, et al.* SNPs associated with cerebrospinal fluid phospho-tau levels influence rate of decline in Alzheimer's disease. *PLoS genetics* **6**, e1001101 (2010).
94. Peterson, D.*, et al.* Variants in PPP3R1 and MAPT are associated with more rapid functional decline in Alzheimer's disease: the Cache County Dementia Progression Study. *Alzheimer's & dementia : the journal of the Alzheimer's Association* **10**, 366-371 (2014).
95. Sick, E.*, et al.* CD47 update: a multifaceted actor in the tumour microenvironment of potential therapeutic interest. *Br J Pharmacol* **167**, 1415-1430 (2012).
96. Sick, E.*, et al.* Activation of CD47 receptors causes proliferation of human astrocytoma but not normal astrocytes via an Akt-dependent pathway. *Glia* **59**, 308-319 (2011).
97. Murata, T.*, et al.* CD47 promotes neuronal development through Src- and FRG/Vav2-mediated activation of Rac and Cdc42. *The Journal of neuroscience : the official journal of the Society for Neuroscience* **26**, 12397-12407 (2006).
98. Lee, S., Craig, B.T., Romain, C.V., Qiao, J. & Chung, D.H. Silencing of CDC42 inhibits neuroblastoma cell proliferation and transformation. *Cancer letters* **355**, 210-216 (2014).
99. Wilhelmus, M.M.*, et al.* Specific association of small heat shock proteins with the pathological hallmarks of Alzheimer's disease brains. *Neuropathol Appl Neurobiol* **32**, 119-130 (2006).
100. Martins-de-Souza, D.*, et al.* Proteomic analysis of dorsolateral prefrontal cortex indicates the involvement of cytoskeleton, oligodendrocyte, energy metabolism and new potential markers in schizophrenia. *Journal of psychiatric research* **43**, 978-986 (2009).
101. Reed, P.W. & Bloch, R.J. Crystallin-gazing: unveiling enzymatic activity. *Journal of neurochemistry* **118**, 315-316 (2011).
102. Hong, G.S.*, et al.* Gene variations in GSTM3 are a risk factor for Alzheimer's disease. *Neurobiology of aging* **30**, 691-696 (2009).
103. Maes, O.C., Schipper, H.M., Chong, G., Chertkow, H.M. & Wang, E. A GSTM3 polymorphism associated with an etiopathogenetic mechanism in Alzheimer disease. *Neurobiology of aging* **31**, 34-45 (2010).
104. Cunniff, B., Wozniak, A.N., Sweeney, P., DeCosta, K. & Heintz, N.H. Peroxiredoxin 3 levels regulate a mitochondrial redox setpoint in malignant mesothelioma cells. *Redox biology* **3**, 79-87 (2014).
105. Desikan, R.S.*, et al.* Heart fatty acid binding protein and Abeta-associated Alzheimer's neurodegeneration. *Molecular neurodegeneration* **8**, 39 (2013).
106. Guo, L.H., Alexopoulos, P. & Perneczky, R. Heart-type fatty acid binding protein and vascular endothelial growth factor: cerebrospinal fluid biomarker candidates for Alzheimer's disease. *European archives of psychiatry and clinical neuroscience* **263**, 553-560 (2013).
107. Haeckel, A., Ahuja, R., Gundelfinger, E.D., Qualmann, B. & Kessels, M.M. The actin-binding protein Abp1 controls dendritic spine morphology and is important for spine head and synapse formation. *The Journal of neuroscience : the official journal of the Society for Neuroscience* **28**, 10031-10044 (2008).
108. Han, J.*, et al.* HIP-55 is important for T-cell proliferation, cytokine production, and immune responses. *Molecular and cellular biology* **25**, 6869-6878 (2005).
109. Kvartsberg, H.*, et al.* Cerebrospinal fluid levels of the synaptic protein neurogranin correlates with cognitive decline in prodromal Alzheimer's disease. *Alzheimer's & dementia : the journal of the Alzheimer's Association* **11**, 1180-1190 (2015).
110. Tarawneh, R.*, et al.* Visinin-like protein-1: diagnostic and prognostic biomarker in Alzheimer disease. *Annals of neurology* **70**, 274-285 (2011).
111. Braunewell, K.H. The visinin-like proteins VILIP-1 and VILIP-3 in Alzheimer's disease-old wine in new bottles. *Frontiers in molecular neuroscience* **5**, 20 (2012).
112. Yuce-Dursun, B., Danis, O., Demir, S., Ogan, A. & Onat, F. Proteomic changes in the cortex membrane fraction of genetic absence epilepsy rats from Strasbourg. *Journal of integrative neuroscience* **13**, 633-644 (2014).
113. Venkataraman, V., Duda, T., Ravichandran, S. & Sharma, R.K. Neurocalcin delta modulation of ROS-GC1, a new model of Ca(2+) signaling. *Biochemistry* **47**, 6590-6601 (2008).

114. Burgoyne, R.D. & Weiss, J.L. The neuronal calcium sensor family of Ca2+-binding proteins. *The Biochemical journal* **353**, 1-12 (2001).
115. Gozal, Y.M., *et al.* Aberrant septin 11 is associated with sporadic frontotemporal lobar degeneration. *Molecular neurodegeneration* **6**, 82 (2011).
116. Mueller, V.J., Wienisch, M., Nehring, R.B. & Klingauf, J. Monitoring clathrin-mediated endocytosis during synaptic activity. *The Journal of neuroscience : the official journal of the Society for Neuroscience* **24**, 2004-2012 (2004).
117. Poupon, V., *et al.* Clathrin light chains function in mannose phosphate receptor trafficking via regulation of actin assembly. *Proceedings of the National Academy of Sciences of the United States of America* **105**, 168-173 (2008).
118. Laquerriere, A., *et al.* Mutations in CNTNAP1 and ADCY6 are responsible for severe arthrogryposis multiplex congenita with axoglial defects. *Human molecular genetics* **23**, 2279-2289 (2014).
119. Hur, J.Y., *et al.* Identification of novel gamma-secretase-associated proteins in detergent-resistant membranes from brain. *The Journal of biological chemistry* **287**, 11991-12005 (2012).
120. Tashima, S., Shimada, S., Yamaguchi, K., Tsuruta, J. & Ogawa, M. Expression of brain-type glycogen phosphorylase is a potentially novel early biomarker in the carcinogenesis of human colorectal carcinomas. *The American journal of gastroenterology* **95**, 255-263 (2000).
121. Lillpopp, L., *et al.* Prognostic information of glycogen phosphorylase isoenzyme BB in patients with suspected acute coronary syndrome. *The American journal of cardiology* **110**, 1225-1230 (2012).
122. Alexander-Kaufman, K. & Harper, C. Transketolase: observations in alcohol-related brain damage research. *The international journal of biochemistry & cell biology* **41**, 717-720 (2009).
123. Morales, D.M., *et al.* Cerebrospinal fluid levels of amyloid precursor protein are associated with ventricular size in post-hemorrhagic hydrocephalus of prematurity. *PloS one* **10**, e0115045 (2015).
124. Morales, D.M., *et al.* Alterations in protein regulators of neurodevelopment in the cerebrospinal fluid of infants with posthemorrhagic hydrocephalus of prematurity. *Molecular & cellular proteomics : MCP* **11**, M111.011973 (2012).
125. Wang, W., *et al.* Role of a neural cell adhesion molecule found in cerebrospinal fluid as a potential biomarker for epilepsy. *Neurochemical research* **37**, 819-825 (2012).
126. Kriebel, M., Wuchter, J., Trinks, S. & Volkmer, H. Neurofascin: a switch between neuronal plasticity and stability. *The international journal of biochemistry & cell biology* **44**, 694-697 (2012).
127. Anlar, B. & Gunel-Ozcan, A. Tenascin-R: role in the central nervous system. *The international journal of biochemistry & cell biology* **44**, 1385-1389 (2012).
128. Zhang, G., Neubert, T.A. & Jordan, B.A. RNA binding proteins accumulate at the postsynaptic density with synaptic activity. *The Journal of neuroscience : the official journal of the Society for Neuroscience* **32**, 599-609 (2012).
129. Woo, H.I., *et al.* Effect of genetic polymorphisms on therapeutic response and clinical outcomes in pancreatic cancer patients treated with gemcitabine. *Pharmacogenomics* **13**, 1023-1035 (2012).
130. Akum, B.F., *et al.* Cypin regulates dendrite patterning in hippocampal neurons by promoting microtubule assembly. *Nature neuroscience* **7**, 145-152 (2004).
131. Shaytan, A.K., Landsman, D. & Panchenko, A.R. Nucleosome adaptability conferred by sequence and structural variations in histone H2A-H2B dimers. *Current opinion in structural biology* **32**, 48-57 (2015).
132. Tian, L., *et al.* Decreased expression of cathepsin D in monocytes is related to the defective degradation of amyloid-beta in Alzheimer's disease. *Journal of Alzheimer's disease : JAD* **42**, 511-520 (2014).
133. Lehri-Boufala, S., *et al.* New roles of glycosaminoglycans in alpha-synuclein aggregation in a cellular model of Parkinson disease. *PloS one* **10**, e0116641 (2015).
134. Chao, M.P., *et al.* Calreticulin is the dominant pro-phagocytic signal on multiple human cancers and is counterbalanced by CD47. *Science translational medicine* **2**, 63ra94 (2010).
135. Lin, Q., Cao, Y. & Gao, J. Serum calreticulin is a negative biomarker in patients with Alzheimer's disease. *International journal of molecular sciences* **15**, 21740-21753 (2014).
136. Stemmer, N., *et al.* Generation of amyloid-beta is reduced by the interaction of calreticulin with amyloid precursor protein, presenilin and nicastrin. *PloS one* **8**, e61299 (2013).
137. Lee, Z.H., *et al.* Identification of a brain specific protein that associates with a refsum disease gene product, phytanoyl-CoA alpha-hydroxylase. *Brain research. Molecular brain research* **75**, 237-247 (2000).
138. Gatson, J.W., *et al.* Detection of neurofilament-H in serum as a diagnostic tool to predict injury severity in patients who have suffered mild traumatic brain injury. *Journal of neurosurgery* **121**, 1232-1238 (2014).

139. Neselius, S., Zetterberg, H., Blennow, K., Marcusson, J. & Brisby, H. Increased CSF levels of phosphorylated neurofilament heavy protein following bout in amateur boxers. *PloS one* **8**, e81249 (2013).
140. Kudo, L.C., *et al.* Puromycin-sensitive aminopeptidase (PSA/NPEPPS) impedes development of neuropathology in hPSA/TAU(P301L) double-transgenic mice. *Human molecular genetics* **20**, 1820-1833 (2011).
141. Varga, I., *et al.* Brevican, neurocan, tenascin-C and versican are mainly responsible for the invasiveness of low-grade astrocytoma. *Pathology oncology research : POR* **18**, 413-420 (2012).
142. van Tijn, P., *et al.* Mutant ubiquitin decreases amyloid beta plaque formation in a transgenic mouse model of Alzheimer's disease. *Neurochemistry international* **61**, 739-748 (2012).
143. Chadwick, L., Gentle, L., Strachan, J. & Layfield, R. Review: unchained maladie - a reassessment of the role of Ubb(+1) -capped polyubiquitin chains in Alzheimer's disease. *Neuropathol Appl Neurobiol* **38**, 118-131 (2012).
144. Capurso, C., *et al.* Polymorphisms in glutathione S-transferase omega-1 gene and increased risk of sporadic Alzheimer disease. *Rejuvenation research* **13**, 645-652 (2010).
145. Allen, M., *et al.* Glutathione S-transferase omega genes in Alzheimer and Parkinson disease risk, age-at-diagnosis and brain gene expression: an association study with mechanistic implications. *Molecular neurodegeneration* **7**, 13 (2012).
146. Kageyama, S., *et al.* Gamma-Glutamylcyclotransferase: A Novel Target Molecule for Cancer Diagnosis and Treatment. *BioMed research international* **2015**, 345219 (2015).
147. Kim, S.H. & Lubec, G. Decreased alpha-endosulfine, an endogenous regulator of ATP-sensitive potassium channels, in brains from adult Down syndrome patients. *Journal of neural transmission. Supplementum*, 1-9 (2001).
148. Grunblatt, E. & Riederer, P. Aldehyde dehydrogenase (ALDH) in Alzheimer's and Parkinson's disease. *Journal of neural transmission (Vienna, Austria : 1996)* **123**, 83-90 (2016).
149. Wey, M.C., *et al.* Neurodegeneration and motor dysfunction in mice lacking cytosolic and mitochondrial aldehyde dehydrogenases: implications for Parkinson's disease. *PloS one* **7**, e31522 (2012).
150. Orosz, F., Olah, J. & Ovadi, J. Triosephosphate isomerase deficiency: facts and doubts. *IUBMB life* **58**, 703-715 (2006).
151. Brown, A.M., *et al.* Substantial linkage disequilibrium across the dihydrolipoyl succinyltransferase gene region without Alzheimer's disease association. *Neurochemical research* **29**, 629-635 (2004).
152. Havik, B., *et al.* DCLK1 variants are associated across schizophrenia and attention deficit/hyperactivity disorder. *PloS one* **7**, e35424 (2012).
153. Khalouei, S., Chow, A.M. & Brown, I.R. Stress-induced localization of HSPA6 (HSP70B') and HSPA1A (HSP70-1) proteins to centrioles in human neuronal cells. *Cell stress & chaperones* **19**, 321-327 (2014).
154. Beaman, G.M., Dennison, S.R., Chatfield, L.K. & Phoenix, D.A. Reliability of HSP70 (HSPA) expression as a prognostic marker in glioma. *Molecular and cellular biochemistry* **393**, 301-307 (2014).
155. Cron, K.R., *et al.* Proteasome inhibitors block DNA repair and radiosensitize non-small cell lung cancer. *PloS one* **8**, e73710 (2013).
156. Czarnecka, A.M., Campanella, C., Zummo, G. & Cappello, F. Heat shock protein 10 and signal transduction: a "capsula eburnea" of carcinogenesis? *Cell stress & chaperones* **11**, 287-294 (2006).
157. Kong, P., *et al.* Neuroprotection of MAO-B inhibitor and dopamine agonist in Parkinson disease. *International journal of clinical and experimental medicine* **8**, 431-439 (2015).
158. Rudrabhatla, P., Grant, P., Jaffe, H., Strong, M.J. & Pant, H.C. Quantitative phosphoproteomic analysis of neuronal intermediate filament proteins (NF-M/H) in Alzheimer's disease by iTRAQ. *FASEB journal : official publication of the Federation of American Societies for Experimental Biology* **24**, 4396-4407 (2010).
159. Martinez-Morillo, E., *et al.* Neurofilament medium polypeptide (NFM) protein concentration is increased in CSF and serum samples from patients with brain injury. *Clinical chemistry and laboratory medicine* **53**, 1575-1584 (2015).
160. Zuo, X., *et al.* Exo70 interacts with the Arp2/3 complex and regulates cell migration. *Nature cell biology* **8**, 1383-1388 (2006).
161. Tedeschi, A., *et al.* RANBP1 localizes a subset of mitotic regulatory factors on spindle microtubules and regulates chromosome segregation in human cells. *Journal of cell science* **120**, 3748-3761 (2007).
162. Cheong, H.S., *et al.* Association of RANBP1 haplotype with smooth pursuit eye movement abnormality. *American journal of medical genetics. Part B, Neuropsychiatric genetics : the official publication of the International Society of Psychiatric Genetics* **156b**, 67-71 (2011).

163. Chen, W., van der Kamp, M.W. & Daggett, V. Structural and dynamic properties of the human prion protein. *Biophysical journal* **106**, 1152-1163 (2014).
164. Dorey, A., *et al.* Association of cerebrospinal fluid prion protein levels and the distinction between Alzheimer disease and Creutzfeldt-Jakob disease. *JAMA neurology* **72**, 267-275 (2015).
165. Peters, C., Espinoza, M.P., Gallegos, S., Opazo, C. & Aguayo, L.G. Alzheimer's Abeta interacts with cellular prion protein inducing neuronal membrane damage and synaptotoxicity. *Neurobiology of aging* **36**, 1369-1377 (2015).
166. Nosrati, N., Kapoor, N.R. & Kumar, V. DNA damage stress induces the expression of ribosomal protein S27a gene in a p53-dependent manner. *Gene* **559**, 44-51 (2015).
167. Woodman, P.G. Biogenesis of the sorting endosome: the role of Rab5. *Traffic (Copenhagen, Denmark)* **1**, 695-701 (2000).
168. Weimer, J.M., *et al.* A BAC transgenic mouse model to analyze the function of astroglial SPARCL1 (SC1) in the central nervous system. *Glia* **56**, 935-941 (2008).
169. Turtoi, A., *et al.* Sparc-like protein 1 is a new marker of human glioma progression. *Journal of proteome research* **11**, 5011-5021 (2012).
170. Strobl-Wildemann, G., *et al.* Novel GDI1 mutation in a large family with nonsyndromic X-linked intellectual disability. *American journal of medical genetics. Part A* **155a**, 3067-3070 (2011).
171. Huang, B., *et al.* Fibroblast growth factors preserve blood-brain barrier integrity through RhoA inhibition after intracerebral hemorrhage in mice. *Neurobiology of disease* **46**, 204-214 (2012).
172. Li, J., *et al.* Increased Expression of Rac1 in Epilepsy Patients and Animal Models. *Neurochemical research* **41**, 836-843 (2016).
173. Gant, J.C., *et al.* FK506-binding protein 1b/12.6: a key to aging-related hippocampal Ca2+ dysregulation? *European journal of pharmacology* **739**, 74-82 (2014).
174. Lleo, A., *et al.* Apotopes and the biliary specificity of primary biliary cirrhosis. *Hepatology (Baltimore, Md.)* **49**, 871-879 (2009).
175. Cheng, T.L., *et al.* Identification and characterization of the mitochondrial targeting sequence and mechanism in human citrate synthase. *Journal of cellular biochemistry* **107**, 1002-1015 (2009).
176. Zhou, B., *et al.* Tumor suppressor candidate gene, NDRG2 is frequently inactivated in human glioblastoma multiforme. *Molecular medicine reports* **10**, 891-896 (2014).
177. Nakajima, J., *et al.* De novo EEF1A2 mutations in patients with characteristic facial features, intellectual disability, autistic behaviors and epilepsy. *Clinical genetics* **87**, 356-361 (2015).
178. Byun, H.O., *et al.* Cathepsin D and eukaryotic translation elongation factor 1 as promising markers of cellular senescence. *Cancer research* **69**, 4638-4647 (2009).
179. Guttman, O., *et al.* Acute-phase protein alpha1-anti-trypsin: diverting injurious innate and adaptive immune responses from non-authentic threats. *Clinical and experimental immunology* **179**, 161-172 (2015).
180. Hunt, J.M. & Tuder, R. Alpha 1 anti-trypsin: one protein, many functions. *Current molecular medicine* **12**, 827-835 (2012).
181. Crosnier, C., *et al.* Basigin is a receptor essential for erythrocyte invasion by Plasmodium falciparum. *Nature* **480**, 534-537 (2011).
182. Shishkin, S.S., Eremina, L.S., Kovalev, L.I. & Kovaleva, M.A. AGR2, ERp57/GRP58, and some other human protein disulfide isomerases. *Biochemistry. Biokhimiia* **78**, 1415-1430 (2013).
183. Li, J. & Lee, A.S. Stress induction of GRP78/BiP and its role in cancer. *Current molecular medicine* **6**, 45-54 (2006).
184. Delpino, A. & Castelli, M. The 78 kDa glucose-regulated protein (GRP78/BIP) is expressed on the cell membrane, is released into cell culture medium and is also present in human peripheral circulation. *Bioscience reports* **22**, 407-420 (2002).
185. Michaelis, E.K., *et al.* Neuronal Glud1 (glutamate dehydrogenase 1) over-expressing mice: increased glutamate formation and synaptic release, loss of synaptic activity, and adaptive changes in genomic expression. *Neurochemistry international* **59**, 473-481 (2011).
186. Burbaeva, G., *et al.* Glutamate metabolizing enzymes in prefrontal cortex of Alzheimer's disease patients. *Neurochemical research* **30**, 1443-1451 (2005).
187. Chambliss, K.L., Gray, R.G., Rylance, G., Pollitt, R.J. & Gibson, K.M. Molecular characterization of methylmalonate semialdehyde dehydrogenase deficiency. *Journal of inherited metabolic disease* **23**, 497-504 (2000).
188. Millard, S.P., *et al.* Association of cerebrospinal fluid Abeta42 with A2M gene in cognitively normal subjects. *Neurobiology of aging* **35**, 357-364 (2014).

189. Bhamra, M.S. & Ashton, N.J. Finding a pathological diagnosis for Alzheimer's disease: are inflammatory molecules the answer? *Electrophoresis* **33**, 3598-3607 (2012).
